# Broad-spectrum biodegradation of aliphatic and aliphatic-aromatic polyesters by *Papiliotrema laurentii* isolated from locust frass

**DOI:** 10.3389/fmicb.2026.1909125

**Published:** 2026-08-25

**Authors:** Sihyun An, Hang-Yeon Weon, Jae-Hyung Ahn, Joon-hui Chung, Dayeon Kim, Young-Joon Ko, Jehyeong Yeon, Jae-Cheol Lee

**Affiliations:** 1 Agricultural Microbiology Division, National Institute of Agricultural Sciences, Wanju, Republic of Korea; 2 Division of Biotechnology, College of Environmental and Bioresource Sciences, Jeonbuk National University, Iksan, Jeonbuk, Republic of Korea; 3 Department of Environmental Engineering, School of Architecture, Civil and Environmental Engineering, Mokpo National University, Muan, Republic of Korea

**Keywords:** biodegradable plastics, mulch film, *Papiliotrema laurentii*, partial biotransformation, terephthalic acid

## Abstract

Biodegradable aliphatic and aliphatic-aromatic polyesters, such as poly(butylene adipate-co-terephthalate) (PBAT), polylactic acid (PLA), polycaprolactone (PCL), polybutylene succinate (PBS), and polyhydroxyalkanoates (PHA), are increasingly used as sustainable alternatives to petrochemical plastics. However, their depolymerization outside industrial composting facilities is often incomplete. This study characterized *Papiliotrema laurentii* strain 62UF-13, isolated from migratory locust frass, for broad-spectrum polyester hydrolysis. Emulsion assays demonstrated hydrolytic activity across all five polymers, with PCL and PBS showing the highest clearance rates. Solid-film assays revealed substantial gravimetric mass loss of PCL, PLA, and PHA cast films, whereas a commercial PBAT–PLA mulch film in minimal medium, underwent progressive fragmentation/disintegration, as assessed by the remaining film area. Incubation with the PBAT–PLA film was accompanied by the release of adipic acid (49.60 mg/L, week 1) and terephthalic acid (maximum 21.62 mg/L, week 4), followed by a decrease to 0.26 mg/L by week 8, coinciding with the emergence of putative 3,4-dihydroxymandelic acid and a putative acetylated derivative. Scanning electron microscopy (SEM) revealed pronounced pitting and erosion, while Fourier-transform infrared (FTIR) spectroscopy and differential scanning calorimetry (DSC) indicated ester-bond scission and changes in crystallinity/melting behavior. Whole-genome sequencing identified eight candidate polyesterases, including cutinases and esterases, with ≥ 60% amino acid identity to known hydrolases active on PCL, PBS, PHA, and PLA. This study is the first report of *P. laurentii* degrading a broad range of aliphatic and aliphatic-aromatic polyesters, including partial biotransformation of terephthalate moieties from PBAT. Integration of phenotypic assays and genomic evidence positions *P. laurentii* 62UF-13 as a viable biocatalyst for decentralized management of biodegradable plastic waste under mild environmental conditions.

## 1 Introduction

The massive global production of synthetic plastics and their inherent resistance to degradation have led to significant ecological and human health risks (Chamas et al., 2020; Galloway and Lewis, 2016; Geyer et al., 2017). Moreover, with global recycling rates remaining low (Leal Filho et al., 2025), even biodegradable plastics can fragment into microplastics due to incomplete degradation, potentially exacerbating environmental contamination rather than mitigating it (Zhu and Wang, 2020). To address these challenges, biodegradable aliphatic and aliphatic–aromatic polyesters, including poly(butylene adipate-co-terephthalate) (PBAT), polylactic acid (PLA), polycaprolactone (PCL), poly(butylene succinate) (PBS), and microbially derived polyhydroxyalkanoates (PHA), are increasingly used as sustainable alternatives (Sander et al., 2023). While these polymers can undergo biological conversion, such as mineralization, under controlled composting conditions (Samir et al., 2022), their biodegradation outside industrial facilities is often inconsistent and incomplete (Lin et al., 2025). Additionally, although bio-based polymers may lower the carbon footprint in the production phase, this advantage does not guarantee effective degradation in natural environments (Folino et al., 2020). For instance, PLA exhibits a semi-crystalline structure that impedes enzymatic attack, allowing it to persist for years in soil or marine environments (Nikolaivits et al., 2022). In agricultural soils, the incomplete breakdown of mulch films (e.g., PBAT–PLA blends) is particularly problematic, as residual microplastics and hydrolysis byproducts, such as terephthalic acid (TA) and 1,4-butanediol (BDO), can transiently accumulate in the plastisphere at concentrations sufficient to affect surrounding biota (Hua et al., 2025). Although TA monomers have shown negligible acute toxicity under controlled conditions (Witt et al., 2001), their persistence in soils with low microbial degradation capacity remains a concern (Wolf et al., 2025), underscoring the need for biological strategies that can both depolymerize these materials and metabolize their aromatic residues.

Microbial degradation represents a sustainable approach for the treatment of polyester waste, utilizing the catalytic activity of lipases, cutinases, and other polyester-degrading enzymes that cleave ester bonds (Nikolaivits et al., 2022). A variety of bacteria and fungi have been reported to degrade aliphatic polyesters and, in some cases, aromatic polyesters; however, the range of target polymers and the efficiency of degradation vary substantially among strains. For example, certain *Pseudomonas* and *Bacillus* species can efficiently degrade PCL and PBS (Hu et al., 2024), whereas other microorganisms degrade PLA or PHA to varying extents (Aoki and Tanaka, 2025). Yeasts have been less extensively studied in this field; however, they offer several advantages, including tolerance to low water activity, adaptability to diverse environmental conditions, and robust secretion of extracellular enzymes. Some basidiomycetous yeasts have shown the capability to degrade synthetic polyesters like PBSA and PCL but often fail to decompose recalcitrant polymers such as PLA. For instance, in a recent screening of 21 smut yeast isolates, none exhibited detectable PLA-degrading activity (Aoki and Tanaka, 2025). Moreover, most previous studies have focused on the degradation of a single polymer under elevated temperatures ( > 50°C) (Liu et al., 2015; Wolf et al., 2025), and systematic comparisons of multi-polymer degradation capabilities using the same microbial strain under ambient conditions remain scarce. More critically, the simple depolymerization of complex blends such as PBAT–PLA is insufficient if hazardous aromatic residues, such as TA, are left intact (Hua et al., 2025; Wolf et al., 2025). This limitation underscores the need to identify microorganisms capable of not only disintegrating diverse polyesters but also driving the metabolic transformation of persistent aromatic monomers.

*Papiliotrema laurentii* (formerly *Cryptococcus laurentii*) is a basidiomycetous yeast of the class Tremellomycetes (order Tremellales, family Rhynchogastremaceae). It was formally reclassified from the polyphyletic genus *Cryptococcus* into *Papiliotrema* by Liu et al. (2015), on the basis of an integrated multi-gene phylogenetic analysis of the Tremellomycetes. The species is ubiquitous in the environment and has been recovered from soil, the phyllosphere, decaying plant material, freshwater, and insect-associated niches, reflecting a broad ecological range and tolerance to environmental stress (de Almeida et al., 2022). It is generally considered non-pathogenic and exhibits notable metabolic versatility: it tolerates low water activity and osmotic stress, accumulates intracellular lipids and carotenoid pigments, and secretes a diverse repertoire of extracellular hydrolases. These properties have been exploited for single-cell-oil production, carotenoid biosynthesis, and the biocontrol of postharvest fungal pathogens, as reviewed by de Almeida et al. (2022). Of particular relevance to the present work, Roman et al. (2024) recently discovered, expressed recombinantly, and analyzed a cutinase from *P*. *laurentii*. This enzyme is capable of hydrolyzing both natural substances like cutin and synthetic polyesters, thus providing direct enzymatic evidence of polyester-degrading capabilities in this species. Consistent with this, cutinase-like esterase from phylogenetically related basidiomycetous yeasts, including *Pseudozyma* (*Moesziomyces*) and *Cryptococcus* spp., have been shown to depolymerize aliphatic polyesters such as PBS, PBSA, and PCL, and in some cases PLA (Masaki et al., 2005; Sameshima-Yamashita et al., 2019). Despite these observations, the polyester-degrading capability of *P*. *laurentii* has been demonstrated mainly at the level of individual enzymes. Systematic experimental validation of whole-cell, multi-polymer degradation, together with its capacity to transform aromatic monomers released from polyester blends under environmentally relevant conditions, has not previously been investigated.

To address this critical gap, the present study investigates the broad-spectrum degradation and partial transformation capabilities of *P. laurentii* 62UF-13, isolated from insect excrement. Moving beyond routine reports of polymer-degrading microorganisms, we comprehensively evaluate the strain’s capacity to disintegrate five major biodegradable polyesters and a commercial PBAT–PLA mulch film at ambient temperature (28°C). Crucially, we present evidence for a previously unreported partial biotransformation of the aromatic monomer TA, whose culture-medium concentration declined in parallel with the appearance of a putative product tentatively identified as 3,4-dihydroxymandelic acid (3,4-DHMA). Supported by kinetic analyses and whole-genome sequencing, this study highlights a potential biological pathway for partially transforming emerging plastic contaminants and underscores its significance for the ecological restoration of polluted soil environments.

## 2 Materials and methods

### 2.1 Isolation and identification of strain

The fungal strain used in this study was isolated in 2015 from the excrement of the migratory locust, *Locusta migratoria*. A pure culture of the yeast strain was cultivated in R2A medium at 28°C. Genomic DNA was extracted from cultured cells using a genomic DNA purification kit (Macrogen Inc., Seoul, Korea). The strain was identified by amplification and sequencing of three ribosomal DNA (rDNA) regions, including the 18S rRNA gene, the internal transcribed spacer (ITS) region, and the 26S rRNA gene. The 18S rRNA gene was amplified using primers NS1 (5′-GTAGTCATATGCTTGTCTC-3′) and NS24 (5′-AAACCTTGTTACGACTTTTA-3′), yielding a fragment of 1,633 bp (accession no. PX974616). The ITS region was amplified using primers ITS1 (5′-TCCGTAGGTGAACCTGCGG-3′) and ITS4 (5′-TCCTCCGCTTATTGATATGC-3′), producing a fragment of 497 bp (accession no. OQ600631). The 26S rRNA gene was amplified using primers LR0R (5′-ACCCGCTGAACTTAAGC-3′) and LR7 (5′-TACTACCACCAAGATCT-3′), generating a fragment of 1,293 bp (accession no. PX974617). PCR amplification was performed under the following thermal recycling conditions: initial denaturation at 95°C for 5 min; 31 cycles of 95°C for 30 s, 55°C for 30 s, and 72°C for 90 s; followed by a final extension at 72°C for 10 min. Amplicons were purified using ExoSAP-IT PCR Product Cleanup Reagent (Thermo Fisher Scientific, Waltham, MA, United States). The purified amplicons were sequenced by Macrogen Inc. (Seoul, South Korea). Sequences were compared pairwise using the Basic Local Alignment Search Tool (BLAST) algorithm in the National center for Biotechnology Information (NCBI) database. Phylogenetic trees were constructed based on the combined sequences of the 18S rRNA gene, ITS region, and 26S rRNA gene. Multiple sequence alignments were performed using the CLUSTAL W program (Thompson et al., 1994). Tree topologies were reconstructed using the maximum-likelihood method implemented in the MEGA 11 software package (Tamura et al., 2021), with bootstrap values calculated from 1,000 resamplings. Based on the combined phylogenetic analysis of the 18S rRNA, ITS, and 26S rRNA gene sequences, strain 62UF-13 was identified as *Papiliotrema laurentii* (Supplementary Figure 1). The strain has been deposited in the Korean Agricultural Culture Collection (KACC) under accession number KACC 83076BP, and the corresponding DNA sequences have been deposited in the GenBank database under accession numbers PX974616 (18S rRNA), OQ600631 (ITS region), and PX974617 (26S rRNA).

### 2.2 Degradation of biodegradable plastic emulsion

All chemicals were purchased from Sigma-Aldrich (St. Louis, MO, United States) unless otherwise specified. The polyesters used in this study were PCL (M_*n*_ 10,000, 45,000, and 80,000), PLA (M_*n*_ 40,000), and PHA, all obtained from Sigma-Aldrich. The PHA was poly(3-hydroxybutyrate) (PHB; natural origin, average M_*n*_ ∼ 500,000), the most common member of the polyhydroxyalkanoate family, and is hereafter referred to as PHA. PBAT and PBS were provided by Ihlshin Chemical Co. (Gyeonggi, Korea) and Ancoplastics Co. (Gangwon, Korea), respectively.

After incubation in R2A broth for 16 h at 28°C, cells were harvested, washed three times with phosphate-buffered saline (PBS), and adjusted to an optical density (OD_600_) of 0.1 using minimal salt medium (MSM). The MSM used for biodegradation assays contained R2A broth at a concentration of 0.003 g/L, and its composition was as follows: NaH_2_PO_4_⋅12H_2_O 3.58g, KH_2_PO_4_ 1.361 g, (NH_4_)_2_SO_4_ 0.3 g, MgSO_4_⋅7H_2_O 0.05 g, CaCl_2_⋅H_2_O 0.0058 g; yeast extract, 0.01 g/L (in distilled water); trace element solution, 10 mL; Se/W solution, 1 mL; vitamin solution, 1 mL; agar, 15 g; and distilled water up to 1 L. A 10 μL aliquot of the cell suspension was then inoculated into an emulsified medium containing 0.2% PCL and 0.1% (w/v) each of PBAT, PBS, PHA, and PLA. Cultures were incubated at 28°C for 4 weeks, and the diameter of the clear zone was measured using an optical microscope (KCX-80LA, Optinity, Seoul, South Korea).

### 2.3 Preparation and biodegradation of solid polyester films

Polyester films were prepared by dissolving 1, 3, and 5% (w/v) polyester pellets in chloroform (2.5, 7.5, and 12.5 g in 250 mL chloroform, respectively), followed by solution casting in glass trays using the conventional solvent-casting method (Urbanek et al., 2017). Films with different average molecular weights were prepared at a concentration of 1% (w/v). After solvent evaporation by air drying for 3 days, the films were cut into 2 × 2 cm^2^ pieces and adjusted to weights of 0.02, 0.06, and 0.10 g. The corresponding nominal thicknesses were calculated using Equation (1):


$$h=\frac{m}{\rho \,A}$$ (1)

Where h is the nominal thickness, m is the specimen mass, ρ is the bulk density of the polymer, and A is the planar area of the specimen (4 cm^2^). Reported bulk densities of 1.146, 1.24, and 1.25 g cm^–3^ were used for PCL, PLA, and PHA, respectively (García-Campo et al., 2017). The resulting nominal thicknesses were approximately 44, 131, and 218 μm for the 1, 3, and 5% PCL films, respectively, and approximately 40 μm for the 1% PLA and PHB films. These values are nominal estimates rather than direct thickness measurements. The films were sterilized in 70% ethanol for 15 min, rinsed with distilled water for 1 min, and subsequently exposed to UV light for 30 min as a pretreatment.

Pre-cultured cells were spiked 0.1 (OD_600_) to 20 mL of liquid MSM medium containing film pieces. Liquid cultivation was performed in a shaking incubator at 28°C, and 150 rpm for 3–8 weeks. At designated time points, films were collected, soaked in 2% sodium dodecyl sulfate for 4 h, rinsed with distilled water, air-dried for 2 days, and stored at −4°C for further analysis. All experiments were conducted in triplicate. Uninoculated abiotic controls (identical films, medium, and incubation without yeast) were run in parallel for every polymer at all-time points and were monitored gravimetrically; day 0 controls were additionally used as the baseline for FTIR, DSC, and SEM analyses.

### 2.4 Hydrolysis of PBAT-PLA mulch film

PBAT–PLA commercial mulch film sheets (1 × 1 cm^2^; BATRO, IHLSHIN Chemical, manufactured in 2024) were used for degradation tests in liquid culture. The PBAT–PLA mulch films were incubated in 50 mL of minimal medium supplemented with 1% R2A broth and three agar plugs of strain 62UF-13 at 28°C and 150 rpm for 8 weeks. To evaluate the biodegradability of PBAT-PLA films, one film sheet was sampled weekly, washed with distilled water, and assessed for disintegration by measuring the remaining film area.

### 2.5 Analysis of degradation of structural changes

#### 2.5.1 Mass loss and microbial growth

Film mass loss was monitored by determining the residual mass of the samples at predetermined time points. After removal from the medium, the films were dried to constant weight in a desiccator for 1 h. The percentage mass loss was calculated by comparing the dry mass at a given time (W1) with the initial mass (W0), according to Equation (2).


$$Degradationrate\left(\%\right)=\frac{\left(W\,0-W\,1\right)}{W\,0}\times 100$$ (2)

#### 2.5.2 Fourier transform infrared spectroscopy

FTIR spectroscopy measurements (4,000–650 cm^–1^, 4 cm^–1^ spectral resolution, 4 scans per sample, in duplicate) were performed using a Perkin Elmer Spectrum 100 spectrometer equipped with attenuated total reflectance (ATR) accessory. For PCL films, the FT-IR crystallinity index (CrI_FTIR_) was calculated using Equation (3):


$$C\,r\,I_{\text{FTIR}}=\frac{A_{1294}}{A_{1167}}\times 100$$ (3)

Where A_ν_ represents the absorbance at wavelength ν. The bands at 1,294 and 1,167 cm^–1^ are associated with crystalline and amorphous PCL chain conformations, respectively (Coleman and Zarian, 1979; Khatiwala et al., 2008). Accordingly, CrI_FTIR_ was interpreted as a relative absorbance-ratio index rather than as an absolute crystalline mass fraction (He and Inoue, 2000). The carbonyl index was calculated as A_1726_/A_1466_ following Khatiwala et al. (2008), whereas the hydroxyl index was operationally defined in the present study as A_3435_/A_1465_. Because simple FT-IR band intensity ratios reflect relative spectral chain organization rather than an absolute three-dimensional crystalline fraction (Coleman and Zarian, 1979; He and Inoue, 2000), CrI_FTIR_ was interpreted separately from DSC-derived crystallinity (X_c, DSC_).

#### 2.5.3 Differential scanning calorimetry analysis

thermal properties were investigated using differential scanning calorimetry (DSC), performed with a DSC 40 instrument (TA Instruments, New Castle, DE, United States) over a temperature range of -40 to 200°C under a nitrogen (N_2_) atmosphere at a heating rate of 10°C/min. The melting temperature (T_m_) and enthalpy of melting (△H_m_) were determined from the second heating scan, and the corresponding second-heating △H_m_ values were used to calculate DSC-derived crystallinity (X_*c,DSC*_).

The degree of crystallinity (X_c_) of the PLA, PCL, and PHA phases was calculated using Equation (4).


$$X_{c,D\,S\,C}\left(\%\right)=\frac{△\,H\,m}{△\,H\,0}\times 100$$ (4)

where △H_m_ represents the measured heat of fusion of the sample, and △H_0_ refers to the heat of fusion of a 100% crystalline polymer, taken as 93.6 J/g for PLA (Reit et al., 2024), 139.5 J/g for PCL (Eryildiz et al., 2025), and 145 J/g for PHA (Thellen et al., 2008).

#### 2.5.4 Field emission scanning electron microscopy

The surface morphologies of films at different stages of degradation were observed through FE-SEM (Carl Zeiss, Oberkochen, Germany) at an accelerating voltage of 3 kV.

### 2.6 Identification of hydrolysis products and metabolites

#### 2.6.1 HPLC analysis of monomeric degradation products

Residual monomers formed during the reaction were analyzed using high-performance liquid chromatography (HPLC). Analyses were performed using a PerkinElmer LC 300 HPLC system (PerkinElmer, Waltham, MA, United States) for 1,4-butanediol, adipic acid (AA), and lactic acid (LA), and a Nexera X2 HPLC system (Shimadzu, Kyoto, Japan) for TA. Culture supernatant (450 μL) was collected, and 300 μL was filtered through a PES syringe filter (0.22 μm; GVS) for HPLC analysis of BDO, AA, and LA. The remaining supernatant was mixed with an equal volume of acetonitrile, incubated at 4 °C overnight, and filtered using a PVDF syringe filter (Whatman–GE Healthcare, Pittsburgh, PA, United States) for HPLC analysis of TA.

For the quantification of BDO, AA, and LA, HPLC was equipped with a refractive index (RI) detector (LC 300 RI Detector, PerkinElmer, Waltham, MA, United States) and a cation-exchange column (Aminex HPX-87H, 7.8 × 300 mm, Bio-Rad Laboratories, Hercules, CA, United States). The mobile phase consisted of 5 mM sulfuric acid as the running buffer, with the column oven maintained at 60 °C. Residual BDO, AA, and LA concentrations were quantified using calibration curves in the ranges of 0–2,253 mg/L (*R*^2^ = 1.00), 0–3,654 mg/L (*R*^2^= 1.00), and 0–2,252 mg/L (*R*^2^ = 1.00), respectively.

For TA quantification, HPLC was performed using a PDA detector (JP/Nexera X2, Shimadzu, Kyoto, Japan) and a C18 reversed-phase column (Eclipse XDB, 4.6 × 150 mm, 5 μm particle size; Agilent). The system was operated at a column oven temperature of 40 °C with UV detection at 254 nm. The mobile phase consisted of acetonitrile (solvent A) and 0.1% formic acid (solvent B). Gradient elution was initiated with 90% solvent B and 10% solvent A for 2 min. From 2 to 14 min, solvent A was increased to 100% and maintained for 2 min. From 16 to 18 min, solvent B was returned to 90% and held for 4 min, and this composition was maintained until the end of the run, resulting in a total analysis time of 22 min per sample. Residual TA was quantified using a standard curve ranging from 0 to 41.53 mg L^–1^ (*R*^2^ = 1.00).

Both measurements were performed with a 5 μL injection volume at a flow rate of 0.8 mL min^–1^. The respective peak areas were integrated and quantified using SimplicityChrom™ CDS software (PerkinElmer, Waltham, MA, United States) for the LC 300 system and LabSolutions software (Shimadzu, Kyoto, Japan) for the Nexera X2 system.

#### 2.6.2 Identification of film hydrolysis products

The possible degradation products of PBAT–PLA mulch film hydrolyzed by *P. laurentii* 62UF-13 were identified using liquid chromatography–tandem mass spectrometry (LC–MS/MS). After 8 weeks of cultivation, the culture supernatants were filtered through a PES syringe filter (0.22 μm; Sanford, ME, United States) and analyzed immediately.

Analyses were performed using an Impact II quadrupole time-of-flight mass spectrometer (Bruker Daltonics, Billerica, MA, United States) equipped with an electrospray ionization (ESI) source operating in negative ion mode. Chromatographic separation was achieved using a Bruker Intensity Solo C18 column (2.0 × 100 mm, 2.0μm particle size), maintained at 40°C. The mobile phase consisted of solvent A (0.1% formic acid in deionized water) and solvent B (0.1% formic acid in acetonitrile). The gradient elution began with 95% solvent A and 5% solvent B, and was maintained isocratically for the first 23 min. From 23 to 28 min, the proportion of solvent B was linearly increased to 100%, where it was held constant for 0.5 min. The mobile phase composition was then returned to the initial conditions (95% solvent A and 5% solvent B) at 28.5 min, and maintained for re-equilibration until the end of the run at 35 min, resulting in a total analysis time of 35 min per sample.

The injection volume was 5μL, and the flow rate was 0.6 mL/min. Mass spectra were acquired over an m/z range of 50–1,200, with the following source conditions: capillary voltage, 3,000 V; nebulizing gas pressure, 13 psi; dry gas flow, 10 L/min; dry gas temperature, 150°C; and collision energy, 20–40 eV. Deprotonated molecular ions ([M–H]^–^) were used for fragmentation analysis. Chromatographic peak areas were integrated, and LC–MS/MS raw data were processed using Bruker Compass MetaboScape software (Bruker Daltonik GmbH, Bremen, Germany). MS/MS fragmentation patterns were examined to assign probable intermediate degradation products.

### 2.7 Genome analysis

#### 2.7.1 Genomic DNA extraction and whole-genome sequencing

High-molecular-weight genomic DNA of *Papiliotrema laurentii* strain 62UF-13 was isolated using the MagAttract High-Molecular-Weight (HMW) DNA Kit (Qiagen, Hilden, Germany), in accordance with the manufacturer’s instructions. Whole-genome sequencing was carried out using both the PacBio Sequel IIe platform (Pacific Biosciences, United States) and the Illumina NovaSeq 6000 system (Illumina, United States). For PacBio sequencing, SMRTbell libraries were prepared, and sequencing was performed using a single SMRT Cell. The raw reads were assembled de novo using the SMRT Link pipeline (v11). Illumina short reads were subsequently used for error correction and gap filling during hybrid assembly. The whole-genome sequence data have been deposited in DDBJ/ENA/GenBank under BioProject accession number PRJNA1116867, BioSample accession number SAMN41553245, and Genome accession number JBDXTD000000000.

#### 2.7.2 Genome annotation and functional prediction

Genome annotation was performed using the MAKER genome annotation pipeline. Secreted proteins were predicted using the deep-learning–based program DeepSig (Savojardo et al., 2018). Candidate effector proteins were identified from the predicted secreted proteins using EffectorP 2.0 (Sperschneider et al., 2018). Carbohydrate-active enzymes (CAZymes) were predicted using the dbCAN2 meta server (Yin et al., 2012; Zhang et al., 2018) (accessed June 9, 2021).^1^ Proteins predicted as CAZymes by either DIAMOND or Hotpep were further classified based on HMMER results. Secondary metabolite biosynthetic gene clusters were identified using antiSMASH (v7.1.0) (Blin et al., 2019). Genome visualization was performed using Circos (v0.69–6) (Krzywinski et al., 2009).

#### 2.7.3 Identification of plastic-degrading enzymes

The annotated protein sequences of strain 62UF-13 were compared against the Plastic DB database (Gambarini et al., 2022) to identify candidate enzymes involved in plastic degradation. Enzyme classes of interest included esterases, cutinases, carboxylesterases, PETases, proteases, and hydrolases. Candidate genes exhibiting ≥ 30% amino acid identity to known plastic-degrading enzymes were selected for further analysis, and polymer-specific annotations (e.g., PBS, PBSA, PCL, PBAT, PET, and nylon) were recorded.

### 2.8 Statistical analyses

All experiments were performed in triplicate. Data are presented as the mean ± standard deviation (SD). Statistical significance was assessed using two-way analysis of variance (ANOVA), followed by Tukey’s multiple comparisons test. Descriptive statistical analyses and graphical representations were generated using Microsoft Excel 2016 (Microsoft Corp., Redmond, WA, United States) and GraphPad Prism (v 11.0, GraphPad Software, United States). *P* < 0.05 was considered statistically significant, with significance levels denoted as *p < 0.05, **p < 0.01, and ***p < 0.001.

## 3 Results and discussion

### 3.1 Polymer emulsion degradation

**FIGURE 1:**
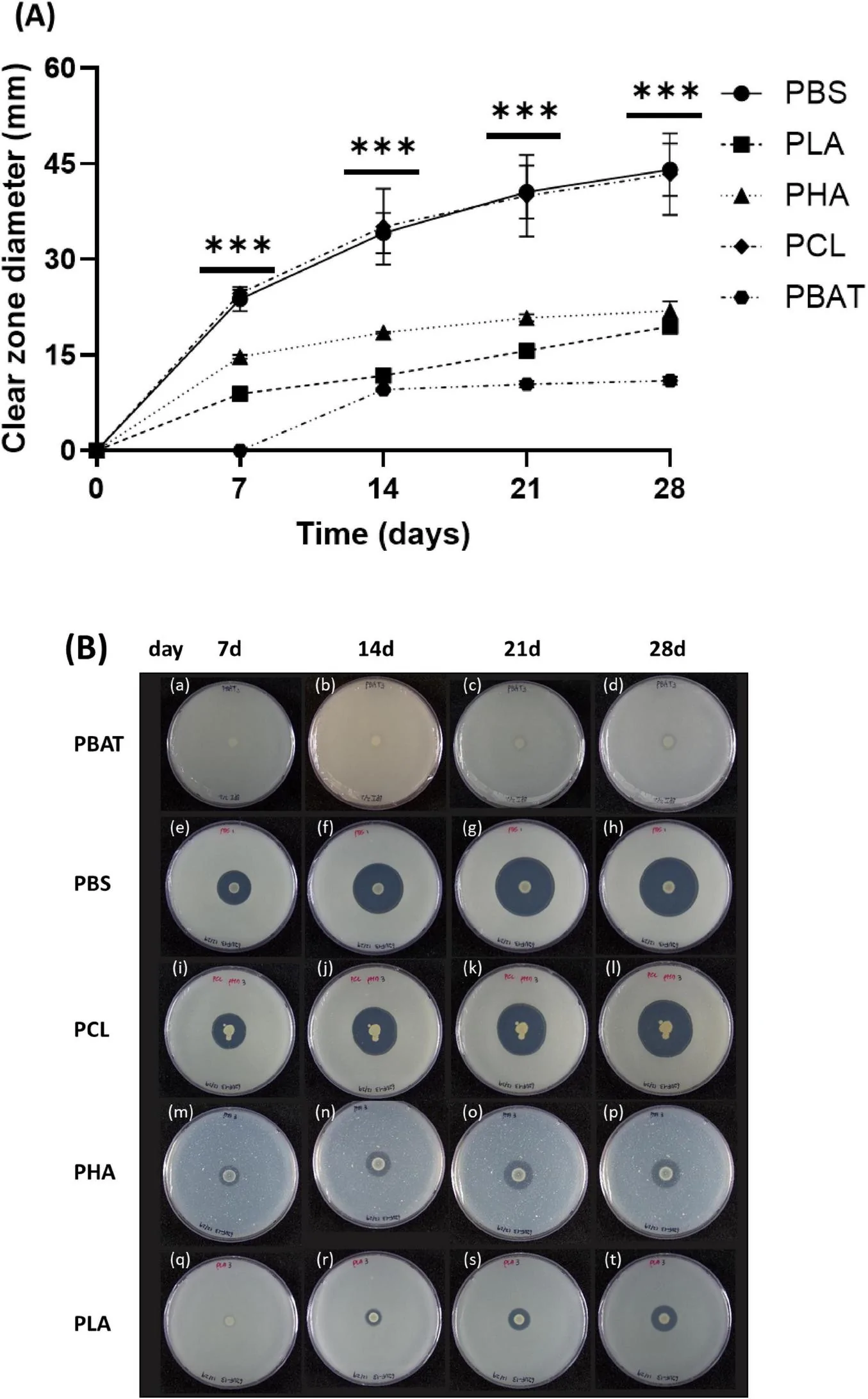
Hydrolytic activity toward emulsified polyesters (PBAT, PBS, PCL, PHA, and PLA). **(A)** Time-dependent changes in clear zone diameter (mm) measured over 28 days, indicating the hydrolytic degradation capacity of the strain. Data are presented as mean ± SD. Statistical significance was determined by two-way ANOVA with Tukey’s *post-hoc* test. PBS and PCL showed no significant difference (ns) at any time point, whereas PHA exhibited significantly smaller clear zones compared to both PBS and PCL at all-time points (***p < 0.001). **(B)** Representative images of clear zone formation on agar plates containing different polyester emulsions at 7, 14, 21, and 28 days. *p < 0.05, **p < 0.01, ***p < 0.001.

As shown in Figure 1, *P. laurentii* 62UF-13 exhibited degradation activity against all five tested biodegradable polyester emulsions, namely PBAT, PLA, PCL, PBS, and PHA. When each polymer was provided as the sole carbon source in minimal medium, clear zones were observed on all inoculated plates over a 28 day incubation period. PCL and PBS reached the 15-mm clear-zone threshold by day 4, PHA by approximately day 7, and PLA by approximately day 21, whereas PBAT remained below this threshold at day 28 (10.97 ± 0.49 mm, as shown in Supplementary Table 1). Because the emulsion clear-zone assay reports secreted extracellular hydrolytic activity rather than complete depolymerization, these times reflect the onset and relative rate of enzymatic attack toward the emulsified polymers and do not, in themselves, establish complete polymer degradation or mineralization. Clear-zone diameter is also constrained by enzyme and hydrolysis-product diffusion within the agar and therefore should not be expected to scale linearly with gravimetric mass loss measured in liquid-film assays. Quantitative degradation was therefore assessed separately using the solid-film mass-loss assays (section 3.2).

These differences in clear-zone expansion kinetics are likely attributed to variations in susceptibility of polymers to hydrolysis. PCL, a low-melting, semi-crystalline polymer, is readily hydrolyzed by lipases, whereas PLA, owing to its high crystallinity and stereocomplex structure, is more resistant to enzymatic degradation (Krumov et al., 2025; Shalem et al., 2024). Nevertheless, the ability of *P. laurentii* strain 62UF-13 to achieve substantial clear-zone formation on the PLA emulsion under relatively mild conditions is noteworthy. This finding highlights the extensive depolymerase repertoire of the yeast, as PLA typically requires specific enzymes or elevated temperatures for efficient biodegradation. The formation of clear zones, including those observed for the more recalcitrant PLA and the aliphatic-aromatic copolyester PBAT, aligns with the release of extracellular enzymes that break down and dissolve the emulsified polyester substrates.

### 3.2 Polyester film degradation

Following confirmation of activity on emulsified substrates, *P. laurentii* 62UF-13 was evaluated for its ability to degrade solid polymer films, including PCL, PLA, and PHA casting films, as well as a commercial PBAT–PLA mulch film. All films were incubated in minimal salt medium for several weeks, with uninoculated films serving as controls.

**FIGURE 2:**
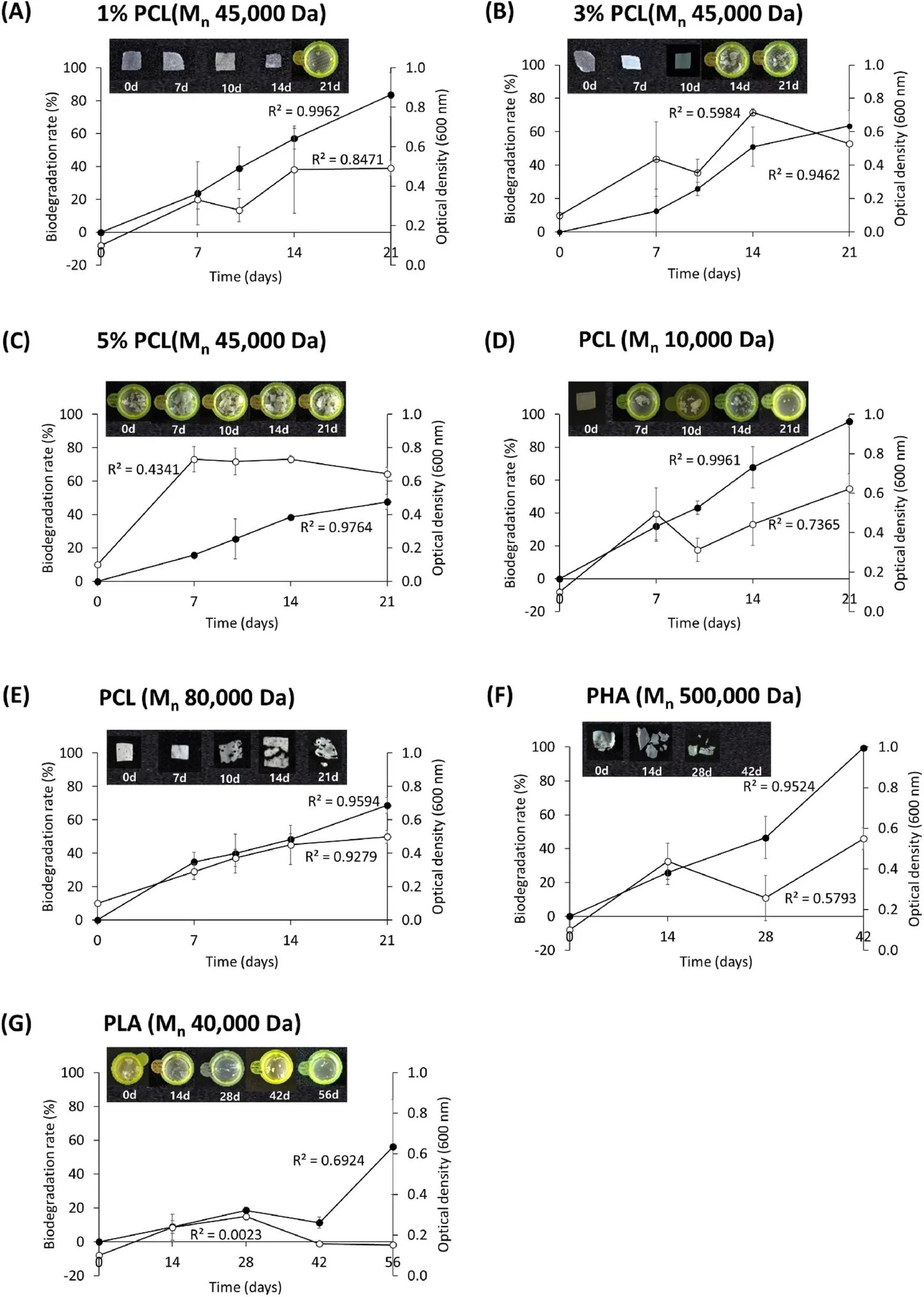
Biodegradation of solid polyester films by strain 62UF-13 in liquid culture. **(A–C)** Mass loss of PCL films with varying concentrations (1, 3, 5%). **(D,E)** Mass loss of PCL films with varying molecular weights (Mn 10k, 80k). **(F)** Approximately complete gravimetric mass loss of the PHA film (99.5%) by day 42 (G) Partial degradation (56%) of PLA film over 56 days. Lines with open circles (-°-) indicate optical density (OD600) representing microbial growth, and lines with filled circles (-∙-) indicate biodegradation rate (%).

The strain exhibited high degradation activity against PCL films of varying concentrations and molecular weights, as well as against PHA and PLA films. After 21 days (Figures 2A–E), mass loss of 1, 3, and 5% PCL films (M_*n*_ 45,000 Da) reached 84 ± 13.3, 64 ± 9.3, and 48 ± 4.5%, respectively, accompanied by a 4.2-fold increase in biomass relative to the initial inoculum. When comparing PCL films of different molecular weights (M_*n*_ 10,000, 45,000, and 80,000 Da), degradation rates were 96 ± 3.3, 84 ± 13.3, and 69 ± 4.8%, respectively, indicating more rapid degradation of lower-molecular-weight films. The molecular weight of the polymer emerged as a critical factor influencing the degradation rate. When comparing polycaprolactone (PCL) films of identical thickness (1% cast) but differing molecular weights (Mn 10,000, 45,000, and 80,000 Da), lower-molecular-weight PCL exhibited significantly faster degradation, with mass loss within 21 days reaching 96 ± 3.3% for M_*n*_ 10,000, compared to 84 ± 13.3% for M_*n*_ 45,000 and 69 ± 4.8% for M_*n*_ 80,000. High-molecular-weight PHA (M_*n*_ 500,000 Da) exhibited complete mass loss within 42 days, whereas PLA (M_*n*_ 40,000 Da) exhibited 56 ± 3.2% mass loss after 56 days (Figures 2F,G). First-order rate constants (k) were 2.3 × 10^–2^ day^–1^ (*R*^2^ = 1.00) for PHA and 1.5 × 10^–2^ day^–1^ (*R*^2^ = 0.62) for PLA, indicating that PHA degraded approximately 1.5-fold faster than PLA. The corresponding uninoculated abiotic controls exhibited low mass loss (0–8%, predominantly 0%) across all polymers and sampling times (Supplementary Table 2), while the day-0 control films served as the spectroscopic, thermal, and morphological baselines. Compared with the low mass loss observed in the abiotic controls, the substantially greater mass loss of the inoculated films (Table 1), together with the corresponding FTIR, DSC, and SEM changes, suggests that microbial activity was the predominant contributor to the observed polymer alterations. However, because time-resolved instrumental analyses were not performed for the abiotic controls, possible contributions from abiotic hydrolysis, swelling, or physical aging cannot be completely excluded.

**TABLE 1:** First-order rate constants (k) and mass loss of solid polyester films allowing degradation by *Papiliotrema laurentii* strain 62UF-13.

Film type	Concentration (%)	Molecular weight (Mn)	Final mass loss (%)	First-order rate constant (k, day^–1^)
PCL	1	45,000	84.0 ± 13.3% (21 days)	6.0 × 10^–2^ (*R*^2^ = 0.97)
PCL	3	45,000	64.0 ± 9.3% (21 days)	5.4 × 10^–2^ (*R*^2^ = 0.93)
PCL	5	45,000	48.0 ± 4.5% (21 days)	3.2 × 10^–2^ (*R*^2^ = 0.98)
PCL	1	10,000	96.0 ± 3.3% (21 days)	8.3 × 10^–2^ (*R*^2^ = 0.91)
PCL	1	80,000	69.0 ± 4.8% (21 days)	5.4 × 10^–2^ (*R*^2^ = 0.98)
PHA	1	500,000	App. 100% (42 days)	2.3 × 10^–2^ (*R*^2^ = 1.00)
PLA	1	40,000	56.0 ± 3.2% (56 days)	1.5 × 10^–2^ (*R*^2^ = 0.62)

Data are presented as mean ± SD (*n* = 3). PCL, polycaprolactone; PHA, polyhydroxyalkanoates; PLA, polylactic acid.

**FIGURE 3:**
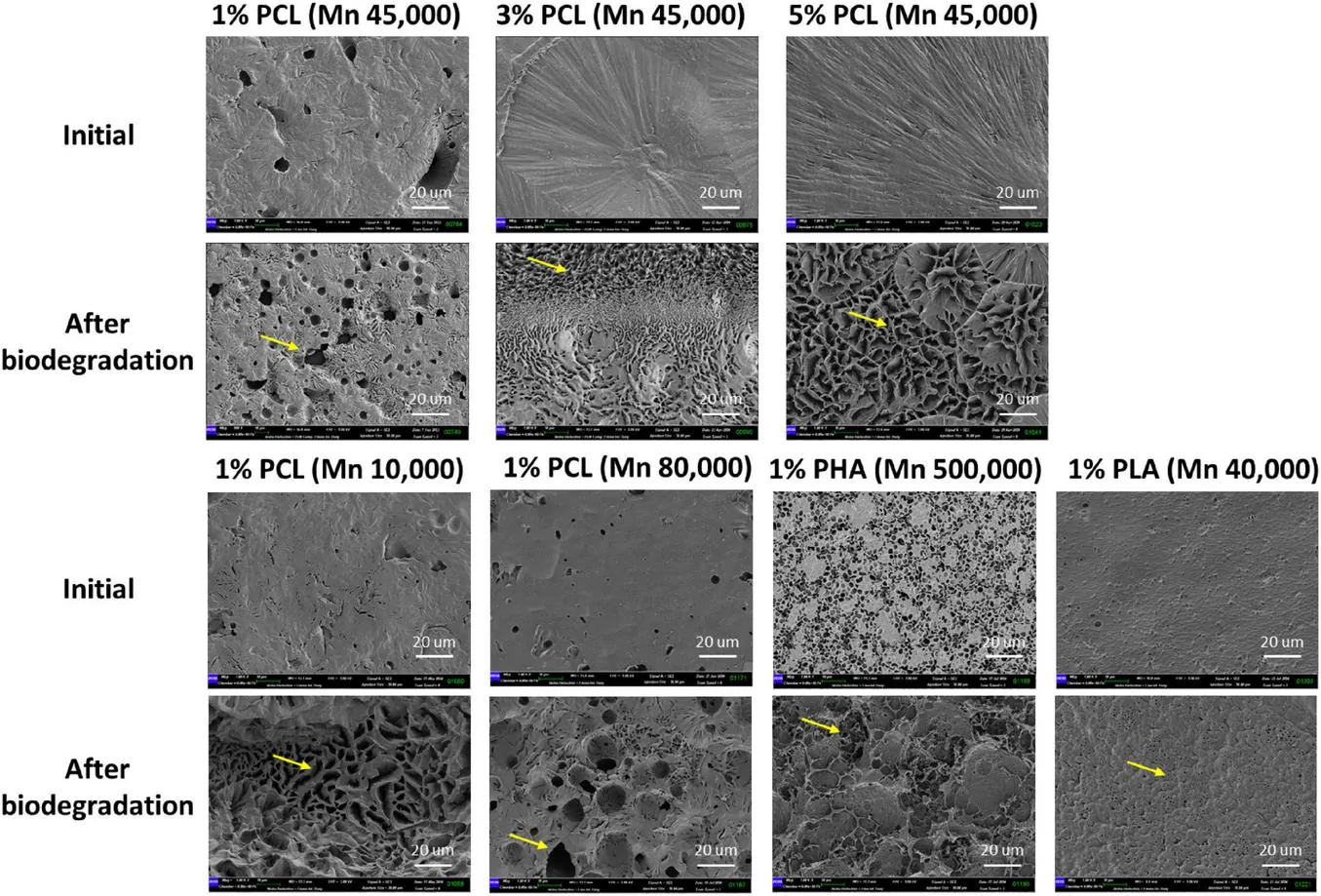
Scanning electron microscopy (SEM) images of polyester films before and after biodegradation by *P. laurentii* 62UF-13. The figure is divided into two panels to compare different degradation factors. (Upper panel) Effect of PCL concentration: Columns represent 1, 3, and 5% PCL (Mn = 45,000). (Lower panel) Effect of molecular weight and polymer type: Columns represent 1% PCL (Mn 10,000 vs. 80,000), 1% PHA, and 1% PLA. Rows: In both panels, the top row displays the initial surface morphology (day 0), while the bottom row presents the surface after biodegradation. Scale bars represent 20 μm. Yellow arrows indicate signs of surface degradation, such as cracks, pits, and structural deterioration. Representative fields are shown for each condition; day 0 panels serve as uninoculated controls. Polymers assessed only as emulsions (PBS) or as commercial mulch film (PBAT) were not cast as solid films and are not included here.

**FIGURE 4:**
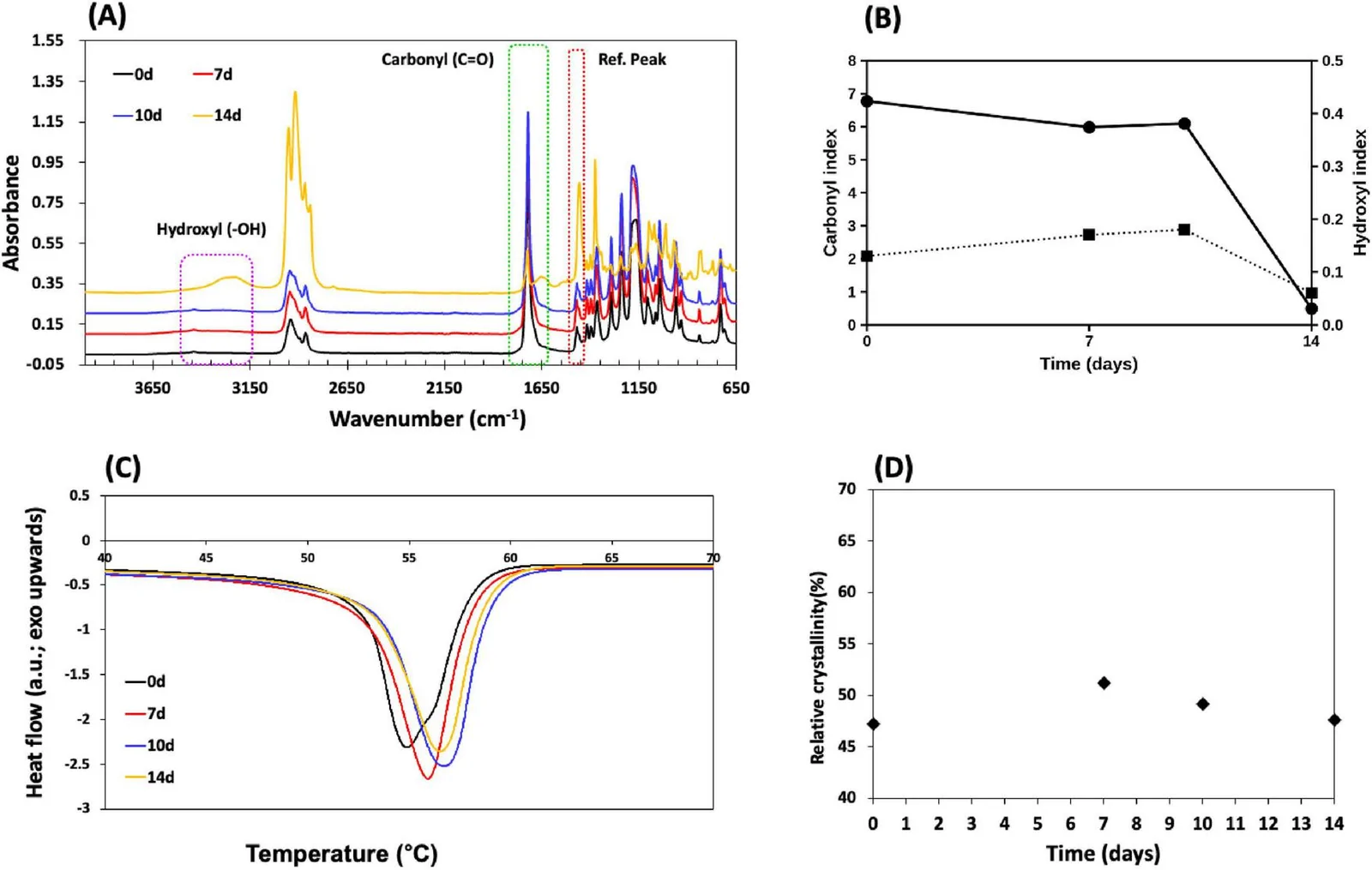
Chemical and thermal analysis of 1% PCL (Mn 45,000) films during biodegradation. **(A)** FT-IR spectra showing functional group changes over 14 days. Dotted boxes indicate the hydroxyl (purple), carbonyl (green), and reference (red) peaks. **(B)** Changes in Carbonyl and Hydroxyl indices calculated from FT-IR absorbance ratios. **(C)** Differential Scanning Calorimetry (DSC) melting curves (endotherms) of PCL films obtained from the second heating scan at a rate of 10°C/min. **(D)** Changes in relative crystallinity (%) over time calculated from the enthalpy of fusion (ΔHf). The initial increase in crystallinity at day 7 suggests the preferential degradation of amorphous regions.

The SEM sampling scheme was designed to examine the two variables evaluated in relation to PCL degradation: casting concentration and the corresponding nominal film thickness (Figures 3A–C), and number-average molecular weight (M_*n*_; Figures 3D,E). Representative fields for each solvent-cast polymer type (PCL, PHA, and PLA; Figures 3A–G) were included, with each post-incubation image paired with its corresponding day-0 baseline. PBS and PBAT were not included in the solvent-cast-film SEM series because single-polymer cast films were not prepared for these polymers. PBS was evaluated only in the emulsion assay, whereas PBAT was examined as an emulsified substrate and as a component of the commercial PBAT–PLA mulch film. SEM micrographs (Figure 3) revealed distinct surface morphological changes in PCL, PHA, and PLA films following biodegradation. For PCL films with a constant molecular weight (M_*n*_ 45,000) but varying thicknesses (A–C: 1, 3, and 5%), the extent of degradation decreased with increasing film thickness. The 1% film (A) exhibited pronounced surface damage, characterized by large, irregular cavities and extensive crack propagation. The 3% film (B) displayed smaller cavities and shorter cracks, whereas the 5% film (C) exhibited a more compact crack network with fewer and smaller cavities, indicating reduced surface erosion. The influence of molecular weight was also evident under constant thickness conditions. The 1% PCL film with Mn 10,000 (D) showed a highly roughened surface with large coalesced voids, whereas the 1% PCL film with Mn 80,000 (E) exhibited a smoother surface with isolated shallow pits and limited cracking, suggesting increased resistance to erosion at higher molecular weight. In contrast, the PHA film (F; 1% M_*n*_ 500,000) exhibited extensive surface etching and widespread delamination, forming interwoven channels and peeling layers across large areas. The PLA film (G; 1% M_*n*_ 40,000) showed only localized pits and short fissures, with most of the surface remaining intact, consistent with its slower degradation kinetics. ATR-FTIR analysis (Figures 4A,B, and Supplementary Figure 2) showed that 1% PCL films (M_*n*_ 45,000 Da) exhibited a decrease in the carbonyl peak (1,720 cm^–1^) and an increase in the hydroxyl peak (3,400 cm^–1^) over 0–14 days, consistent with ester bond hydrolysis; the corresponding carbonyl, hydroxyl, and relative FT-IR crystallinity indices for the 1% PCL films are summarized in Table 2. The relative FT-IR crystallinity index increased from 46 to 59%, suggesting preferential degradation of amorphous regions. Similar trends were observed for 3 and 5% PCL films, as well as for PCL films with M_*n*_ values of 10,000 and 80,000 Da (Supplementary Table 3). PLA and PHA films also exhibited decreases in C = O absorbance and increases in –OH absorbance upon extended incubation. DSC analysis revealed condition-dependent changes in the DSC-derived degree of crystallinity (X_c,DSC_) (Figures 4C,D and Supplementary Figure 3). The 1% PCL film exhibited an initial increase followed by a decline, whereas the 3% film showed a nonmonotonic profile and the 5% film exhibited an overall decreasing trend. These distinct temporal profiles may reflect differences in the relative contributions of amorphous-region removal and crystalline-domain disruption among the film conditions. Mechanistically, this biphasic profile is characteristic of enzymatic surface erosion of semicrystalline polyesters: the loosely packed amorphous regions, including interlamellar amorphous material, are more accessible to secreted depolymerases and are hydrolyzed first, transiently enriching the residual crystalline fraction and raising the apparent crystallinity (Spyros et al., 1997). After the amorphous phase is largely consumed, hydrolysis advances onto the exposed lamellar crystallite surfaces, reducing the absolute crystalline mass and, consequently, the measured crystallinity. The continuous crystallinity decrease observed for PHA (Table 3) indicates concurrent erosion of amorphous and crystalline domains, whereas the initial increase seen for PCL and PLA reflects a more pronounced amorphous-first stage. This polymer-dependent pattern is broadly consistent with Skorokhoda et al. (2025), who reported that PHB crystallinity generally decreased with increasing mass loss, whereas PLA crystallinity remained stable or increased depending on the microorganism and incubation conditions. Fitting the PCL mass loss data to a first-order reaction model yielded k values (Table 1 and Supplementary Figure 4A) of 6.0 × 10^–2^ (*R*^2^ = 0.97), 5.4 × 10^–2^ (*R*^2^ = 0.93), and 3.2 × 10^–2^ (*R*^2^ = 0.98) for 1, 3, and 5% PCL (M_*n*_ 45,000 Da) films, respectively. For molecular weight variation, k values (Table 1 and Supplementary Figure 4B) were 8.3 × 10^–2^ day^–1^ (*R*^2^ = 0.91), 6.0 × 10^–2^ day^–1^ (*R*^2^ = 0.97), and 5.4 × 10^–2^ day^–1^ (*R*^2^ = 0.98) for M_*n*_ 10,000, 45,000, and 80,000 Da films, respectively. Regression coefficients describing the effects of concentration and molecular weight were -6.9 × 10^–3^ (*R*^2^ = 0.91) and -4.2 × 10^–3^ (*R*^2^ = 0.90), respectively. The k values (Table 1 and Supplementary Figures 4C,D) for PHA and PLA films were 2.3 × 10^–2^ day^–1^ (*R*^2^ = 1.00) and 1.5 × 10^–2^ day^–1^ (*R*^2^ = 0.62), respectively, confirming faster degradation of PHA relative to PLA. The corresponding biodegradation (mass-loss and microbial-growth) profiles for all films are provided in Supplementary Figure 5.

**TABLE 2:** Time-resolved ATR-FTIR indices for the representative 1% PCL film (Mn 45,000) during biodegradation.

Incubation time (days)	Relative FTIR crystallinity index (A_1294_/A_1167_ × 100)	Carbonyl index (A_1726_/A_1466_)	Hydroxyl index (A_3435_/A_1465_)
Day 0	45.87	6.77	0.13
Day 7	55.63	5.98	0.17
Day 10	55.85	6.09	0.18
Day 14	58.50	0.49	0.06

**TABLE 3:** Second-heating DSC thermal parameters of solid polyester films during biodegradation.

Polymer	Incubation time (day)	T_*m*_ (°C)	Δ H_*m*_ (J/g)	Xc_*DSC*_ (%)
1% PCL (Mn 45,000)	0	54.9	65.9	47.2
	7	55.8	71.4	51.2
	10	56.5	68.5	49.1
	14	56.5	66.4	47.6
3% PCL (Mn 45,000)	0	56.8	56.8	56.0
	7	55.9	76.4	54.7
	10	56.2	75.5	54.1
	14	54.5	86.7	62.1
	21	54.8	62.3	44.7
5% PCL (Mn 45,000)	0	53.9	82.0	58.8
	7	54.8	78.3	56.1
	10	56.1	73.9	53.0
	14	54.8	71.9	51.5
	21	54.7	76.5	54.8
1% PCL (Mn 10,000)	0	53.5	81.4	58.4
	7	54.6	89.6	64.2
	10	54.3	85.7	61.4
1% PCL (Mn 80,000)	0	58.0	64.7	46.4
	7	55.8	63.9	45.8
	10	57.5	68.7	49.2
	14	56.0	66.0	47.3
	21	55.7	72.4	51.9
1% PHA (Mn 500,000)	0	173.3	96.1	66.3
	14	174.3	89.1	61.4
	28	173.2	89.6	61.8
1% PLA (Mn 40,000)	0	172.2	55.0	58.7
	14	173.4	65.0	69.4
	28	173.1	68.0	72.7
	42	172.9	60.9	65.1
	56	173.2	61.5	65.7

Taken together, the apparent gravimetric mass-loss kinetics and SEM observations indicate that film geometry, molecular architecture, solid-state morphology, and polymer chemistry acted jointly. Within the PCL series, the decrease in *k* with increasing casting concentration at fixed M_*n*_ is consistent with the lower surface-area-to-volume ratio of the nominally thicker films and the less extensive surface erosion observed by SEM. At fixed casting conditions, the decrease in *k* with increasing M_*n*_ may reflect M_*n*_-dependent differences in chain entanglement, chain-end density, segmental mobility, and surface organization; however, the present data do not isolate these contributions, and higher M_*n*_ should not be assumed to increase crystallinity. Previous studies likewise showed that PCL enzymatic hydrolysis is affected by molecular weight and terminal-group density in monolayers and by crystallinity and amorphous–lamellar organization in films (Kulkarni et al., 2007; Sekosan and Vasanthan, 2010). Across polymer types, the fitted apparent rate constants followed PCL > PHA (PHB) > PLA (Table 1); because the PLA fit was weaker (*R*^2^ = 0.62), this order should be interpreted descriptively rather than as a comparison of intrinsic depolymerization constants. Moreover, the order cannot be explained by crystallinity alone: PHB had a higher initial DSC crystallinity than PLA (66.3% vs. 58.7%; Table 3) but showed faster mass loss. This comparison points to enzyme–substrate compatibility as an additional factor: PHB is a natural microbial polyester susceptible to extracellular PHA depolymerases (Paloyan et al., 2025), whereas PLA hydrolysis under mesophilic conditions requires compatible PLA-active hydrolases (Karamanlioglu and Robson, 2013; Shalem et al., 2024). The reference 1% PCL film had the lowest initial T_m_ and DSC crystallinity among the three polymer films (54.9 °C and 47.2%, respectively), consistent with its greater susceptibility to surface hydrolysis. The 56% gravimetric mass loss of PLA after 56 days at 28 °C, together with the PLA clear-zone assay and FTIR changes consistent with ester-bond hydrolysis, supports strain-associated extracellular PLA-hydrolyzing activity but does not identify the responsible enzymes or demonstrate mineralization. Because film roughness and wettability were not quantified, their individual contributions cannot be separated in the present study.

### 3.3 PBAT–PLA mulch film degradation and by-products

**FIGURE 5:**
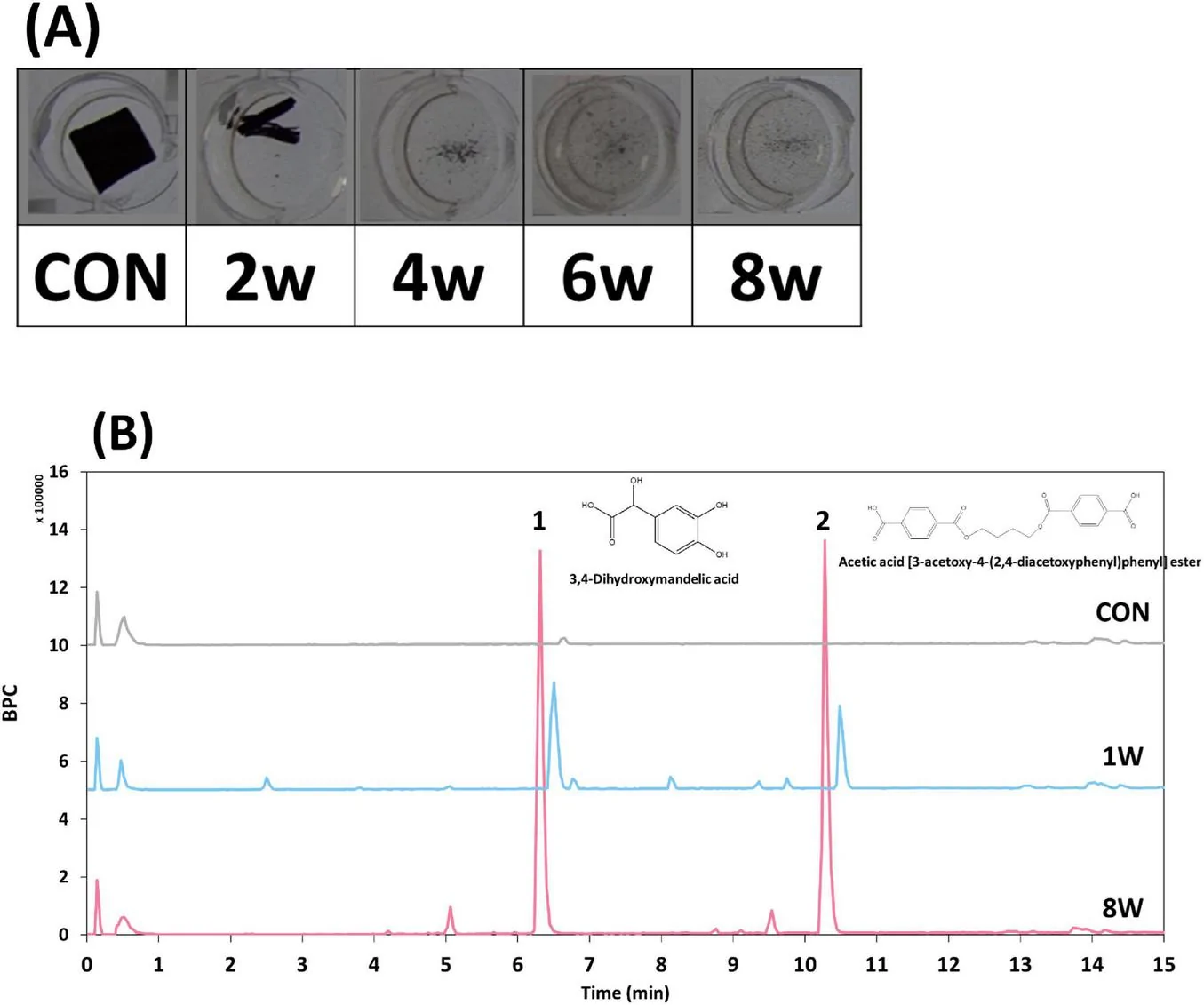
Biodegradation of PBAT-PLA mulch films and identification of metabolic intermediates by *Papiliotrema laurentii* 62UF-13. **(A)** Macroscopic observation of PBAT-PLA mulch film degradation during the 8-week incubation period, showing physical disintegration. **(B)** Identification of degradation products in the culture supernatant after 8 weeks by LC-MS/MS. The detection of putative 3,4-dihydroxymandelic acid (3,4-DHMA) and an acetylated derivative is consistent with transformation of TA via hydroxylation and acetylation pathways rather than simple accumulation.

*P. laurentii* 62UF-13 also exhibited degradation activity toward PBAT–PLA blend mulch films. Film degradation was assessed by the remaining film area and visual disintegration (fragmentation) rather than by gravimetric mass loss. After 2 weeks of incubation the films became brittle and began to fragment, and by 8 weeks they had disintegrated into small pieces. In contrast, the uninoculated PBAT–PLA control films retained a constant mass (0.002 g) and showed no visible disintegration or detectable adipic acid or TA throughout the 8-week incubation. Because PBAT–PLA depolymerization yields both aliphatic (AA, BDO, and LA) and aromatic (TA) monomers, the concentrations of these products in the culture medium were monitored. HPLC analysis showed that AA was detected at 49.60 and 23.48 mg/L during weeks 1 and 2, respectively, but was undetectable thereafter, suggesting rapid assimilation by the yeast. TA was detected at 5.84 mg/L in week 1, increased to 21.62 mg/L by week 4, and declined to 0.26 mg/L by week 8, while remaining undetectable in uninoculated controls (Supplementary Table 4). LC–MS/MS examination of the week-8 samples detected two previously unassigned products (Figure 5B). Based on their accurate-mass measurements and product-ion fragmentation patterns, these products were provisionally assigned as 3,4-dihydroxymandelic acid (3,4-DHMA; retention time, 6.3 min) and acetic acid [3-acetoxy-4-(2,4-diacetoxyphenyl)phenyl] ester (retention time, 10.3 min). These assignments remain tentative because they were not verified using authentic standards or NMR spectroscopy. If these tentative assignments are correct, they would indicate hydroxylation of the aromatic ring and acetylation through further modification. Although complete degradation of TA could not be confirmed, the decrease in its concentration in the culture medium, together with the detection of putative oxidation products, suggests that TA underwent partial transformation rather than simply accumulating in the medium.

A principal finding of this study is the ability of *P. laurentii* 62UF-13 to degrade a commercial PBAT–PLA mulch film using it as the sole carbon source. Unlike previously reported co-culture strategies, in which PLA and PBAT are degraded by different microorganisms (Jia et al., 2021), this yeast strain independently depolymerizes the PBAT–PLA blend. Fragmentation of the film began after 2 weeks and, by the eighth week, the material had disintegrated into small fragments (assessed by remaining film area), indicating that the strain can facilitate film breakdown even at ambient temperature. We note that this endpoint reflects physical disintegration rather than a fully quantitative (gravimetric) measure of polymer degradation. Among the released monomers, the aliphatic component AA was transiently detected but rapidly consumed, suggesting that strain 62UF-13 not only depolymerizes PBAT but also efficiently metabolizes the resulting aliphatic intermediates. In contrast, the aromatic constituent TA has rarely been reported to be metabolized by yeasts. Notably, strain 62UF-13 demonstrated partial conversion capability. TA was first detected in the culture supernatant after 1 week, reached a peak concentration of approximately 22 mg/L by week 4, and subsequently declined to 0.26 mg/L by week 8. At the final stage, two putative derivatives, tentatively assigned as 3,4-dihydroxymandelic acid (3,4-DHMA) and an acetylated derivative, were detected. If confirmed, these putative products would be consistent with hydroxylation of the terephthalate ring, an oxidative modification typically mediated by specialized bacterial dioxygenases (Kincannon et al., 2022). To the best of our knowledge, such oxidative transformation of terephthalate derivatives has not been previously reported in yeasts. The detection of 3,4-DHMA highlights a previously unrecognized oxidative catabolic potential in *P. laurentii*, resembling the initial hydroxylation steps observed in bacterial pathways for aromatic ring cleavage (Wu et al., 2022). Hydroxylation of aromatic intermediates generally enhances their susceptibility to subsequent cleavage reactions. This is exemplified by bacterial pathways in which TA is converted into dihydroxylated intermediates, such as protocatechuate, which then undergo ring fission and are funneled into central metabolism (Sasoh et al., 2006). By analogy, the putative conversion of TA into 3,4-DHMA by strain 62UF-13 may render the aromatic ring more amenable to subsequent cleavage. Collectively, these findings suggest that *P. laurentii* 62UF-13 mediates a partial biotransformation that limits the accumulation of aromatic monomer TA in the medium while potentially expanding its substrate range. This dual capability not only broadens the ecological significance of this yeast but may also reduce the persistence of aromatic residues during mulch film degradation, underscoring its biotechnological potential for sustainable plastic waste management. Whether this transformation lowers toxicity was not assessed here and remains to be verified by dedicated ecotoxicological assays.

### 3.4 Genomic insights into polyester degradation pathways

Whole-genome sequencing of *P. laurentii* strain 62UF-13 yielded a high-quality assembly comprising 15 primary contigs and 42 haplotigs, totaling 19,229,050 bp with a mean GC content of 56.2%. The genome annotation identified 8,471 coding sequences (CDSs), 84 tRNAs, and 26 rRNAs. Supplementary Figure 6 presents a Circos representation of the 15 primary contigs, showing the genomic locations of predicted effector genes, secondary-metabolite biosynthetic gene clusters, and CAZyme-encoding sequences. GC content analysis revealed regions with values above (green) or below (purple) the genome-wide mean, whereas GC skew analysis identified G- and C-biased regions.

Functional annotation using the EggNOG database indicated that the most abundant category was posttranslational modification, protein turnover, and chaperones (category O, 6.0%), followed by carbohydrate transport and metabolism (category G, 5.79%), intracellular trafficking, secretion, and vesicular transport (category U, 4.86%), and translation, ribosomal structure, and biogenesis (category J, 4.81%). Genes of unknown function accounted for 42.7% of all CDSs (3,353 genes), suggesting that a subset may encode uncharacterized hydrolases involved in plastic degradation.

A total of 492 CAZyme-encoding genes were identified in the genome of strain 62UF-13 using the CAZy database. The repertoire comprised 234 glycoside hydrolases (GHs), 145 glycosyltransferases (GTs), 39 carbohydrate-binding modules (CBMs), 32 auxiliary activities (AAs), 31 carbohydrate esterases (CEs), and 11 polysaccharide lyases (PLs) (Supplementary Figure 7). GHs were the most abundant enzyme class and are involved in the hydrolysis of glycosidic bonds. These were followed by GTs and CBMs. This CAZyme distribution is consistent with metagenomic analyses of synthetic plastic-associated communities, including polyethylene (PE), polyethylene terephthalate (PET), and nylon (Zhang et al., 2018).

Comparative analysis against the PlasticDB database identified a diverse set of candidate plastic-degrading enzymes in the genome of strain 62UF-13, predicted to act on a broad range of biodegradable and synthetic polymers, including PBS, PBSA, PCL, PHB, PLA, and PHA (Supplementary Table 5). Esterase genes exhibited up to 65.8% amino acid identity to enzymes from *Pseudozyma antarctica*, which have been previously implicated in the depolymerization of PBS, PBSA, and PCL. Cutinase genes showed 64.1% identity to enzymes from *Cryptococcus* sp., with reported activity against PBS, PCL, PHB, PLA, and PHA (Masaki et al., 2005; Sameshima-Yamashita et al., 2019). The presence of esterase and cutinase genes provides strong genetic support for the potential broad polyester-hydrolyzing capacity of strain 62UF-13. This finding is consistent with recent reports demonstrating that *P. laurentii* possesses cutinase enzymes (Roman et al., 2024).

These genomic predictions were supported by functional assays demonstrating extracellular polymer-degrading activity. Culture supernatants of strain 62UF-13 efficiently hydrolyzed polyester emulsions, producing clear halos indicative of polymer degradation. Clear zones were also observed on agar plates containing emulsified aliphatic polyesters as the sole carbon source, confirming that the strain secretes depolymerases capable of converting solid polymer particles into soluble products. This concordance between genomic potential and observed extracellular activity is consistent with the involvement of the predicted esterase and cutinase genes; however, direct evidence of their expression (e.g., transcriptomic or proteomic analysis) was not obtained in this study and remains to be established. Collectively, these findings establish *P. laurentii* 62UF-13 as a genetically and functionally versatile yeast capable of degrading a broad spectrum of biodegradable plastics and synthetic polymers, thereby positioning it as a promising candidate for biotechnological applications in plastic waste biodegradation and recycling.

### 3.5 Limitations and future perspectives

While strain 62UF-13 exhibited broad and efficient polyester degradation, these results should be interpreted with caution in the context of real environmental application. All degradation experiments in this study were conducted under controlled laboratory conditions, using a single strain in a defined medium. In contrast, natural soils harbor complex and competitive microbial communities, present heterogeneous polymer exposure, and are subject to fluctuating temperature, moisture, oxygen, and nutrient conditions. The high degradation activity observed here should therefore not be directly extrapolated to field environments. A further limitation is analytical rather than environmental. Film thickness was estimated rather than directly measured, and quantitative surface characterization of the films, including wettability (water contact angle) and surface energy, and surface roughness, was beyond the instrumental scope of this study, and such analysis would further clarify the role of microbial attachment in the observed degradation differences. Although the low mass loss observed in the gravimetric abiotic controls (0–8%, predominantly 0%) supports microbial activity as the predominant contributor to the observed degradation, time-resolved FTIR, DSC, and SEM characterization of these controls was not performed because no substantial mass change was observed; such time-course control analyses would further strengthen this attribution and should be included in future studies. It should be noted, however, that the primary aim of this study was to recover and characterize a functional bioresource from an environment in which polyester degradation naturally occurs, as a first step toward its valorization. Building on this isolate, our subsequent objective is either to steer the microbial communities of poorly degrading environments toward enhanced degradation or to construct dedicated, high-efficiency degradation systems. Achieving these objectives will require validation beyond defined laboratory conditions. This validation should employ soil microcosms, non-sterile agricultural soils, and field-scale mulch-film residues within complex matrices, complemented by quantitative surface characterization of the polymer films, which we identify as an essential direction for further studies.

## 4 Conclusion

*Papiliotrema laurentii* 62UF-13 exhibited extensive depolymerization activity across five biodegradable polyesters: PBAT, PLA, PCL, PBS, and PHA in emulsion assays, with additional solid-film degradation demonstrated for PCL, PLA, PHA, and a commercial PBAT–PLA mulch film. Degradation rates were influenced by polymer chemistry, molecular weight, and concentration. Degradation was rapid for the readily biodegradable aliphatic polyesters (PCL, PBS, and PHA), slower for the more recalcitrant aliphatic polyester PLA, and slowest for the aliphatic–aromatic copolyester PBAT. Notably, PHA films underwent approximately complete gravimetric mass loss (99.5%) within 42 days, whereas PLA exhibited 56% mass loss over 56 days. During PBAT–PLA degradation, adipic acid and terephthalic acid were released; putatively identified hydroxylated and acetylated derivatives of terephthalic acid were also detected, consistent with partial transformation of aromatic monomers. Kinetic and spectroscopic analyses revealed preferential attack on amorphous domains, followed by degradation of the crystalline phase. This metabolic versatility, coupled with the ability to transform aromatic monomers, underscores the potential of strain 62UF-13 as a biotechnological tool for composting, soil remediation, and enzymatic recycling of mixed biodegradable plastics.

## Data Availability

The data presented in the study are deposited in the NCBI GenBank repository, accession numbers PX974616 (18S rRNA gene), OQ600631 (ITS region), and PX974617 (26S rRNA gene). The whole-genome sequence is deposited in the DDBJ/ENA/GenBank repository, BioProject accession number PRJNA1116867, BioSample accession number SAMN41553245, and Genome accession number JBDXTD000000000. The strain is deposited in the Korean Agricultural Culture Collection (KACC), accession number KACC 83076BP.

## Footnotes

1
http://bcb.unl.edu/dbCAN2/
