# Supplementary material for: Broad-spectrum biodegradation of aliphatic and aliphatic-aromatic polyesters by Papiliotrema laurentii isolated from locust frass
Source: Front Microbiol. 2026 Aug 25;17:1909125. doi: 10.3389/fmicb.2026.1909125 (PMC13549985; doi:10.3389/fmicb.2026.1909125)
Supplement: Supplementary file 1 [file Supplementary_file_1.pdf]

Supplementary Material

Broad-spectrum biodegradation of aliphatic and aliphatic-aromatic polyesters by *Papiliotrema laurentii* isolated from locust frass

Sihyun An [a, b], Hang-Yeon Weon [a], Jae-Hyung Ahn [a], Joon-hui Chung [a], Dayeon Kim [a], Young-Joon Ko [a], Jehyeong Yeon [a] and Jae-Cheol Lee [c, *]

[a] Agricultural Microbiology Division, National Institute of Agricultural Sciences, Wanju 55365, Republic of Korea

[b] Division of Biotechnology, College of Environmental and Bioresource Sciences, Jeonbuk National University, Iksan, Jeonbuk, 54596, Republic of Korea

[c] Department of Environmental Engineering, School of Architecture, Civil and Environmental Engineering, Mokpo National University, Muan, 58554, Republic of Korea

*Corresponding author: Jae-Cheol Lee (jc.lee@mnu.ac.kr)

Table S1. Biodegradation activity of *Papiliotrema laurentii* strain 62UF-13 on biodegradable polyester emulsions. Data are presented as mean ± SD (n = 3).

Polymer Emulsion	Clear Zone Formation Time (days)	Clear Zone Diameter at 28 days (mm)
PBAT	> 28	10.97±0.49
PLA	~ 21	19.47±0.39
PCL	4	43.37±5.24
PBS	4	44.07±3.36
PHA	7	21.90±1.23

Table S2. Gravimetric mass loss of cast-polymer films under uninoculated abiotic-control and yeast-inoculated conditions during incubation.

Film (Concentration, M_n)	Abiotic-control mass loss (%)	Inoculated-treatment mass loss (%) or outcome
PCL 1% Mn 45,000 (0–21 d)	0, 0, 0, 0, 0	0, 23.7, 38.8, 57.0, 83.7
PCL 3% Mn 45,000 (0–21 d)	0, 1.3, 1.1, 0, 8.2	0, 12.5, 25.8, 51.0, 63.6
PCL 5% Mn 45,000 (0–21 d)	0, 6.8, 0, 0, 0	0, 15.7, 25.4, 38.4, 47.6
PCL 1% Mn 10,000 (0–21 d)	0, 0, 0, 0, 0	0, 31.8, 43.2, 67.8, 95.7
PCL 1% Mn 80,000 (0–21 d)	0, 0, 0, 0.3, 0	0, 34.8, 39.8, 48.3, 68.7
PHA 1% Mn 500,000 (0–42 d)	0, 0, 0	25.8 (14 d), 46.5 (28 d), 99.5 (42 d)
PLA 1% Mn 40,000 (0–56 d)	0, 0, 0, 0.8, 0	8.8 (14 d), 18.7 (28 d), 11.3 (42 d), 56.2 (56 d)

PBAT–PLA mulch (0–8 w)	0 (constant 0.002 g)	fragmentation and disintegration

Table S3. Complete relative FTIR crystallinity index (CrI_{FTIR}) profiles for all PCL conditions during biodegradation

Incubation Time (Days)	1% PCL (M_n 45,000)	3% PCL (M_n 45,000)	5% PCL (M_n 45,000)	1% PCL (M_n 10,000)	1% PCL (M_n 80,000)
Day 0	45.87	40.66	53.18	48.84	43.92
Day 7	55.63	42.05	53.70	52.79	39.85
Day 10	55.85	49.89	48.29	55.30	52.59
Day 14	58.50	52.24	56.04	60.37	61.78
Day 21	-	54.50	51.16	-	-

Table S4. Changes in metabolite concentrations during PBAT–PLA mulch film degradation. Adipic acid and TA remained below the detection limit in the uninoculated controls at all sampling times.

Metabolite (HPLC)	Concentration at Week 1 (mg/L)	Concentration at Week 2 (mg/L)	Concentration at Week 3 (mg/L)	Concentration at Week 4 (mg/L)	Concentration at Week 5 (mg/L)	Concentration at Week 6 (mg/L)	Concentration at Week 7 (mg/L)	Concentration at Week 8 (mg/L)

Adipic acid	49.60	23.48	0	0	0	0	0	0
Terephthalic acid (TA)	5.84	5.92	11.27	21.62	1.08	1.45	0.37	0.26

Table S5. List of predicted plastic-degrading enzyme candidates in the *Papiliotrema laurentii* strain 62UF-13 genome.

Enzyme Family	Predicted Polymer Target(s)	DB Hit ID number	Identity (%)	E-value	Species	Ref
Esterase	PBS	103	65.8	3.90E-90	*Pseudozyma antarctica*	(Sameshima-Yamashita et al., 2019)
	PBSA					
	PCL					
Cutinase	PBS	37	64.1	1.90E-96	*Cryptococcus* sp.	(Masaki et al., 2005)
	PCL					
	PHB					
	PLA					
	PHA					

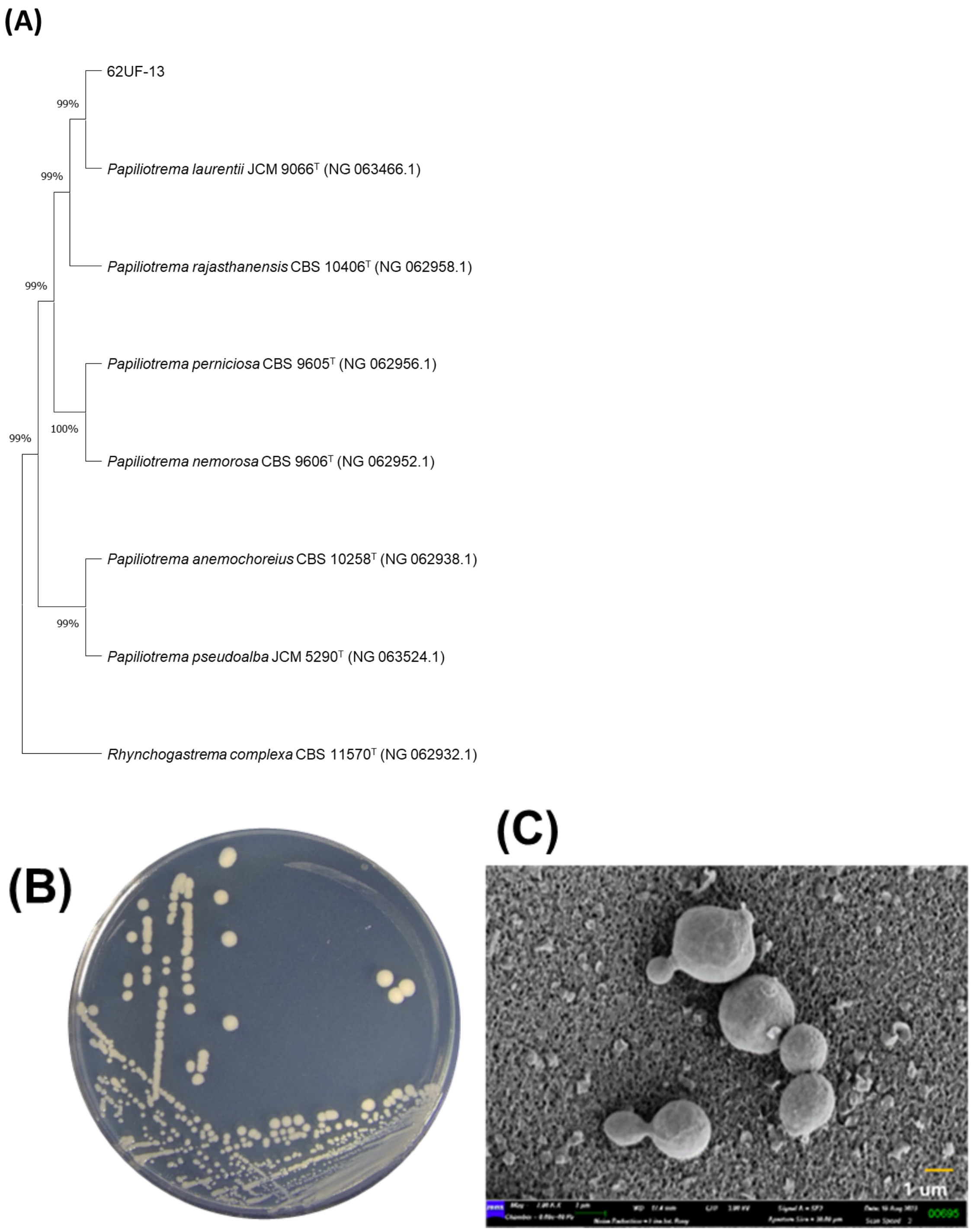

Figure S1. Identification and morphological characterization of *Papiliotrema laurentii* strain 62UF-13. (A) Phylogenetic tree based on concatenated sequences of the 18S rRNA gene, ITS region, and 26S rRNA gene, constructed using the maximum-likelihood method. Strain 62UF-13 (bold) clusters with *P. laurentii* ATCC 18803^T with high bootstrap support. The tree was rooted using *Rhynchogastrema complexa* CBS 11570^T. (B) Colony morphology of strain 62UF-13 grown on R2A agar for 3 days at 28℃, showing mucoid, cream-colored colonies. (C) Scanning

electron micrograph (SEM) showing typical yeast-like cellular morphology and budding reproduction.

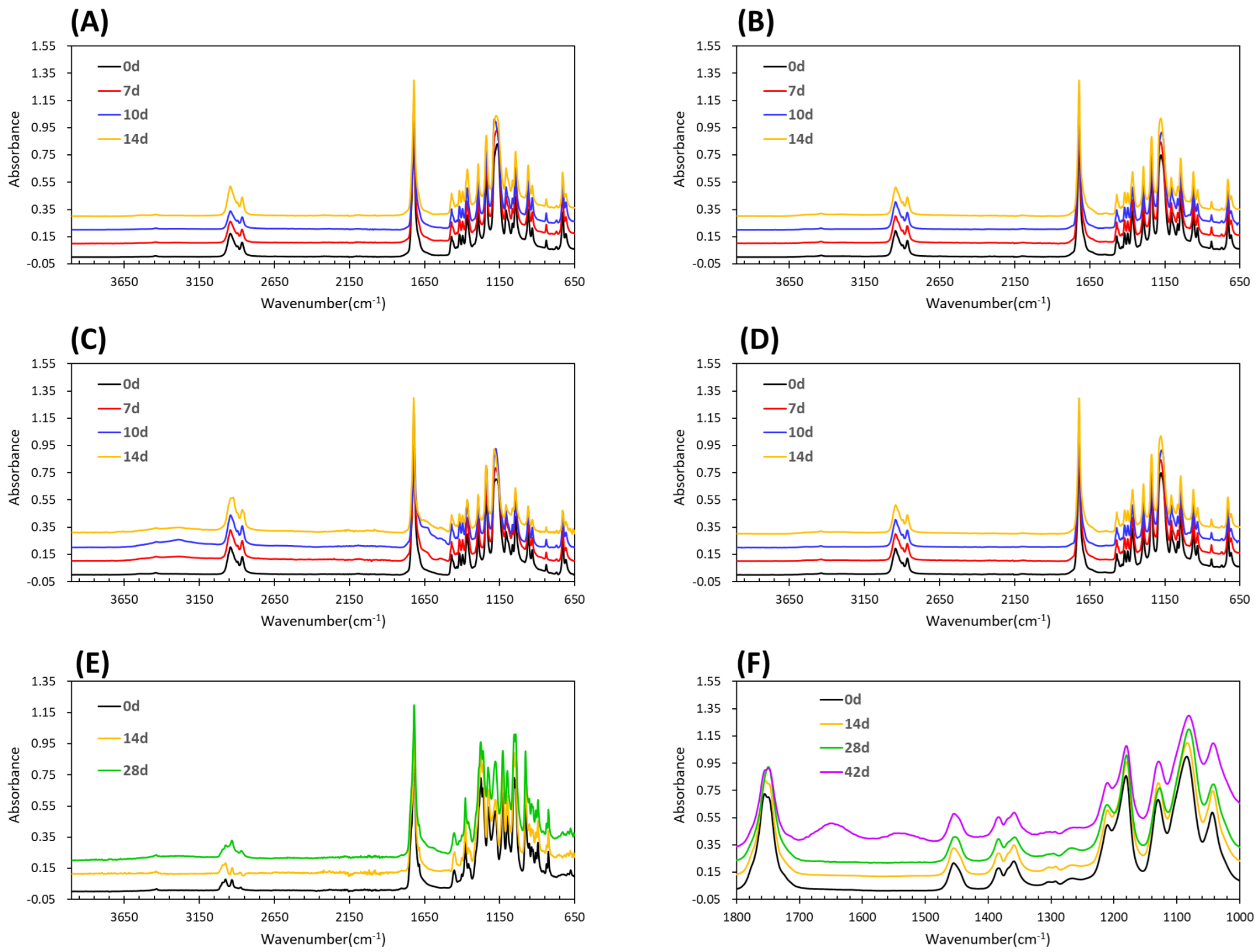

Figure S2. ATR–FTIR spectra of various polyester films during biodegradation. Spectra show chemical changes in films with different properties over time. **(A–B)** PCL films at concentrations of 3% and 5%. **(C–D)** PCL films with molecular weights (M_n) of 10,000 and 80,000 Da. **(E)** PHA (M_n = 500,000) and **(F)** PLA (M_n = 40,000) films. Consistent with the results for 1% PCL in the main text, a reduction in the ester carbonyl peak (1726 cm^{-1}) was observed across all samples, indicating enzymatic hydrolysis.

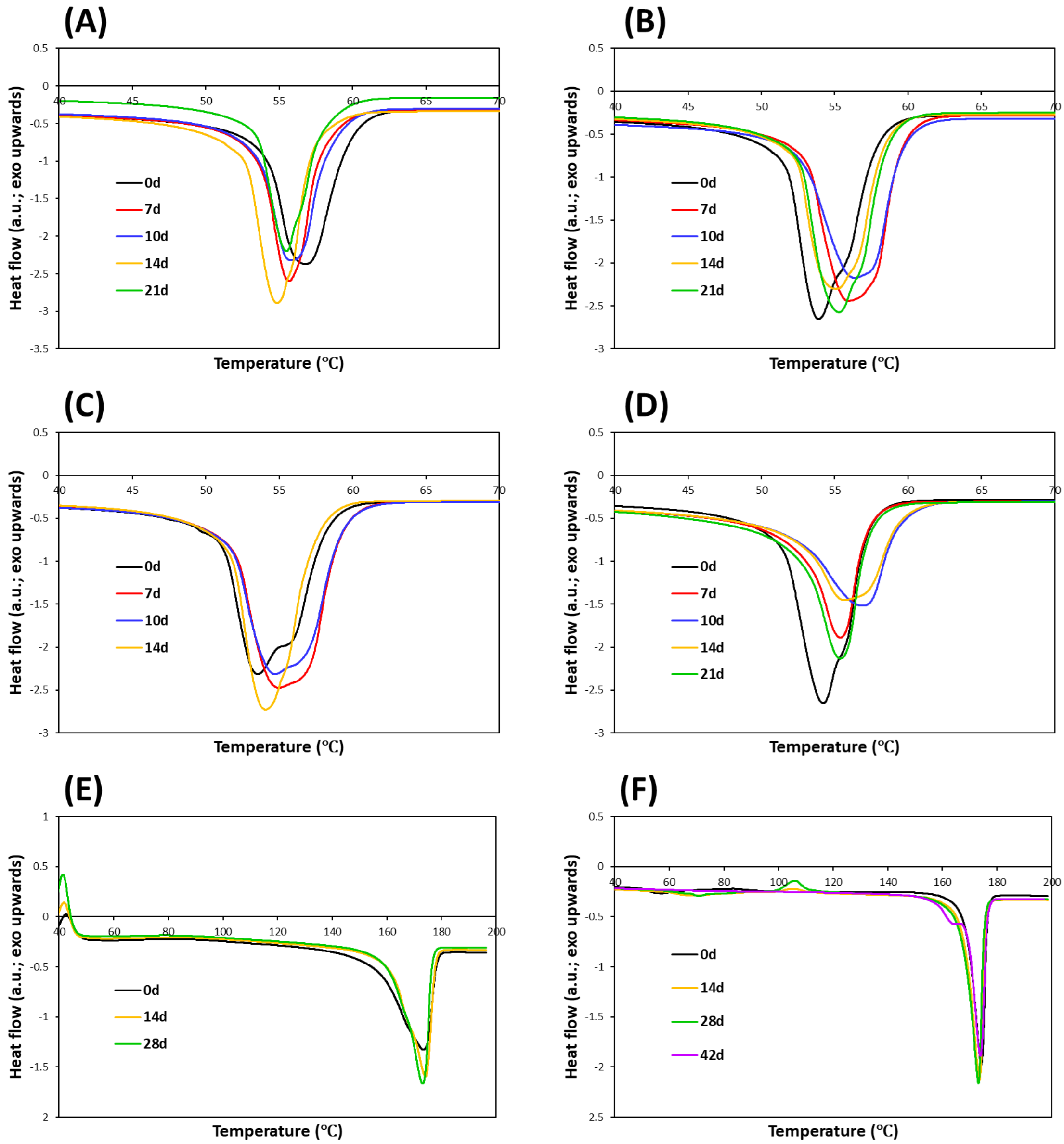

Figure S3. DSC thermograms of polyester films during biodegradation. Changes in melting behavior and heat flow are shown for **(A–B)** PCL films (3%, and 5%), **(C–D)** PCL films (M_n 10,000 and 80,000 Da), **(E)** PHA (M_n = 500,000), and **(F)** PLA (M_n = 40,000) films.

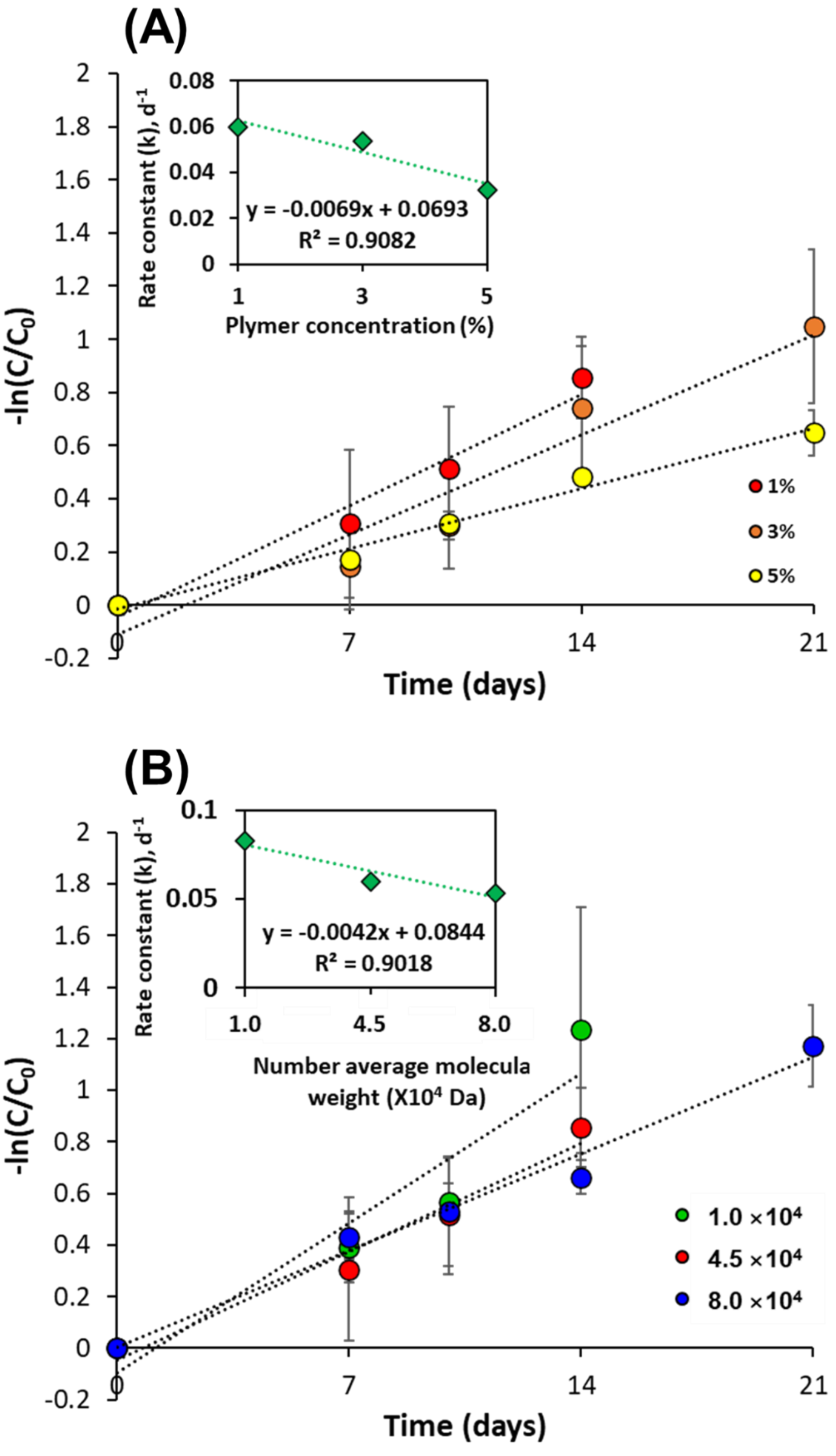
(A)
-ln(C/C0)
Time (days)
Rate constant (k), d-1
Plymer concentration (%)
y = -0.0069x + 0.0693
R² = 0.9082
1%
3%
5%
(B)
-ln(C/C0)
Time (days)
Rate constant (k), d-1
Number average molecula weight (X10⁴ Da)
y = -0.0042x + 0.0844
R² = 0.9018
1.0 ×10⁴
4.5 ×10⁴
8.0 ×10⁴

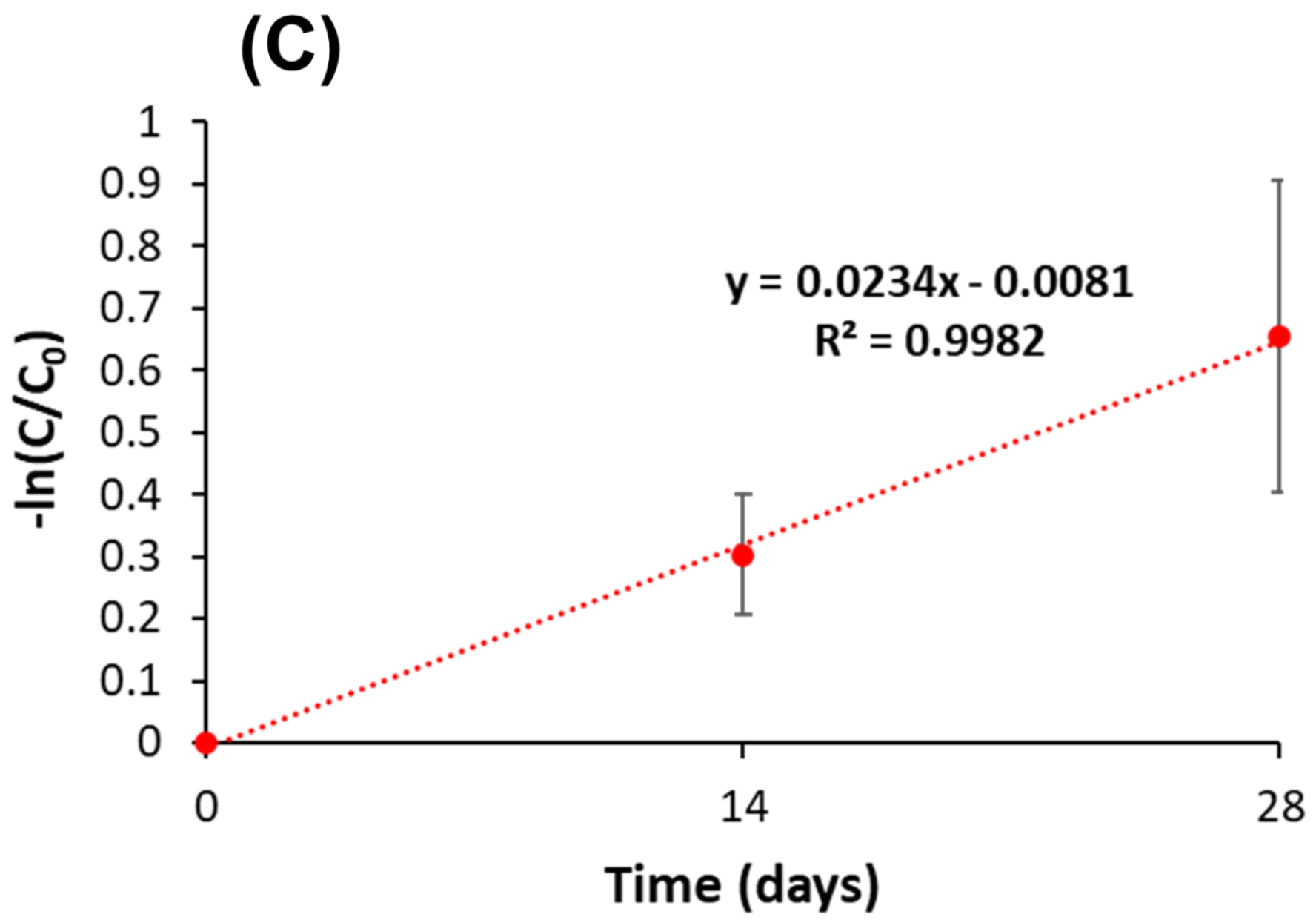

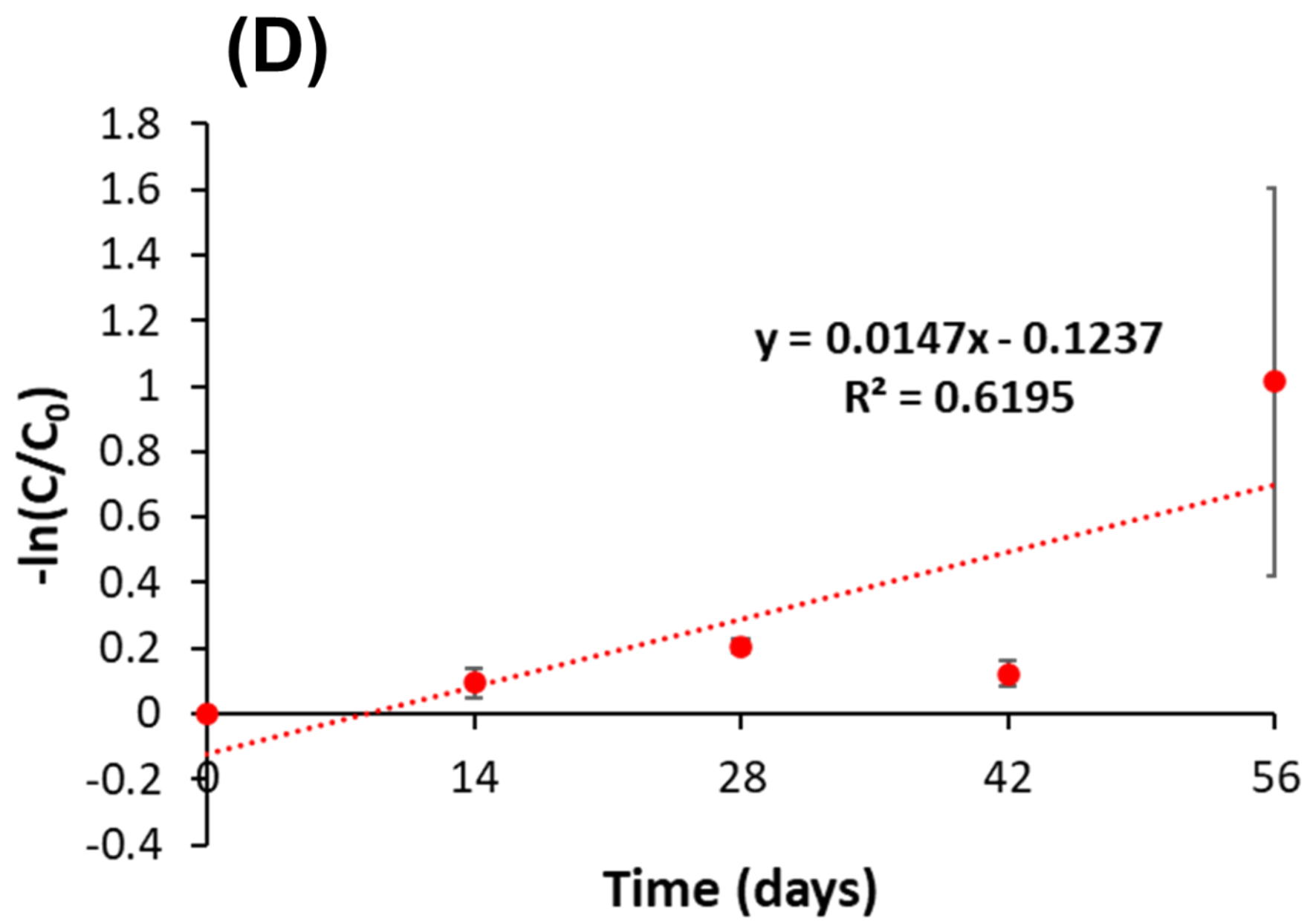

Figure S4. First-order kinetic plots for the biodegradation of polyester films. Linear regression analysis of $-\ln(C/C_0)$ versus time was used to calculate degradation rate constants (*k*). **(A)** Effect of PCL concentration (1%, 3%, and 5%). **(B)** Effect of PCL molecular weight (M_n, 1.0×10^4, 4.5×10^4, 8.0×10^4). **(C)** PHA and **(D)** PLA films. The high coefficients of determination ($R^2 > 0.9$ for most PCL samples) indicate that biodegradation follows first-order kinetics.

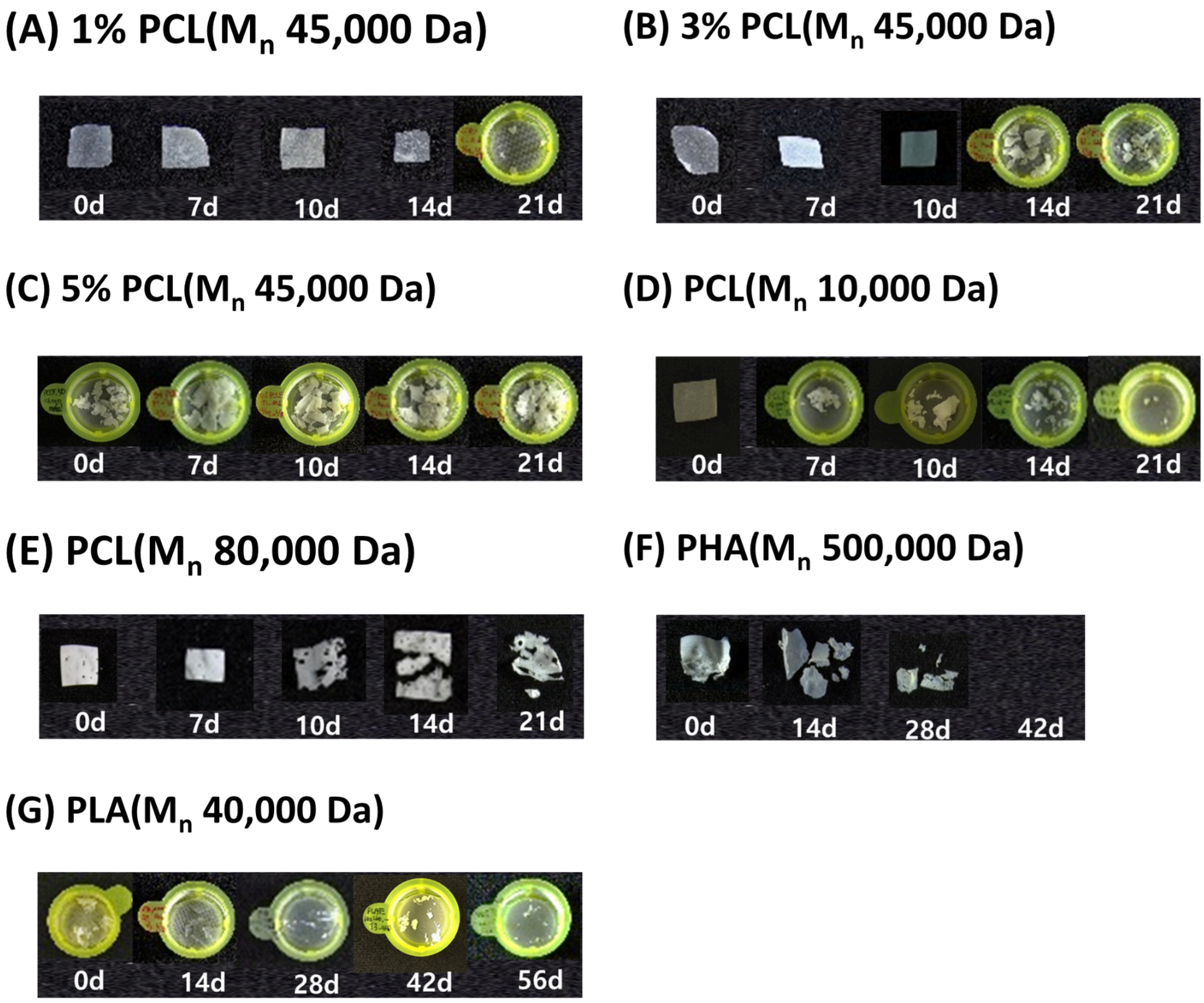

Figure S5. Biodegradation profiles of various polyester films by strain 62UF-13 in liquid culture. (A–C) Mass loss of PCL films (M_n = 45,000) at different concentrations (1%, 3%, and 5%). **(D–E)** Biodegradation of PCL films (1%) with different molecular weights (M_n = 10,000 and 80,000). **(F)** Complete degradation of PHA film within 42 days. **(G)** Partial degradation of PLA film, reaching 56% mass loss over 56 days.

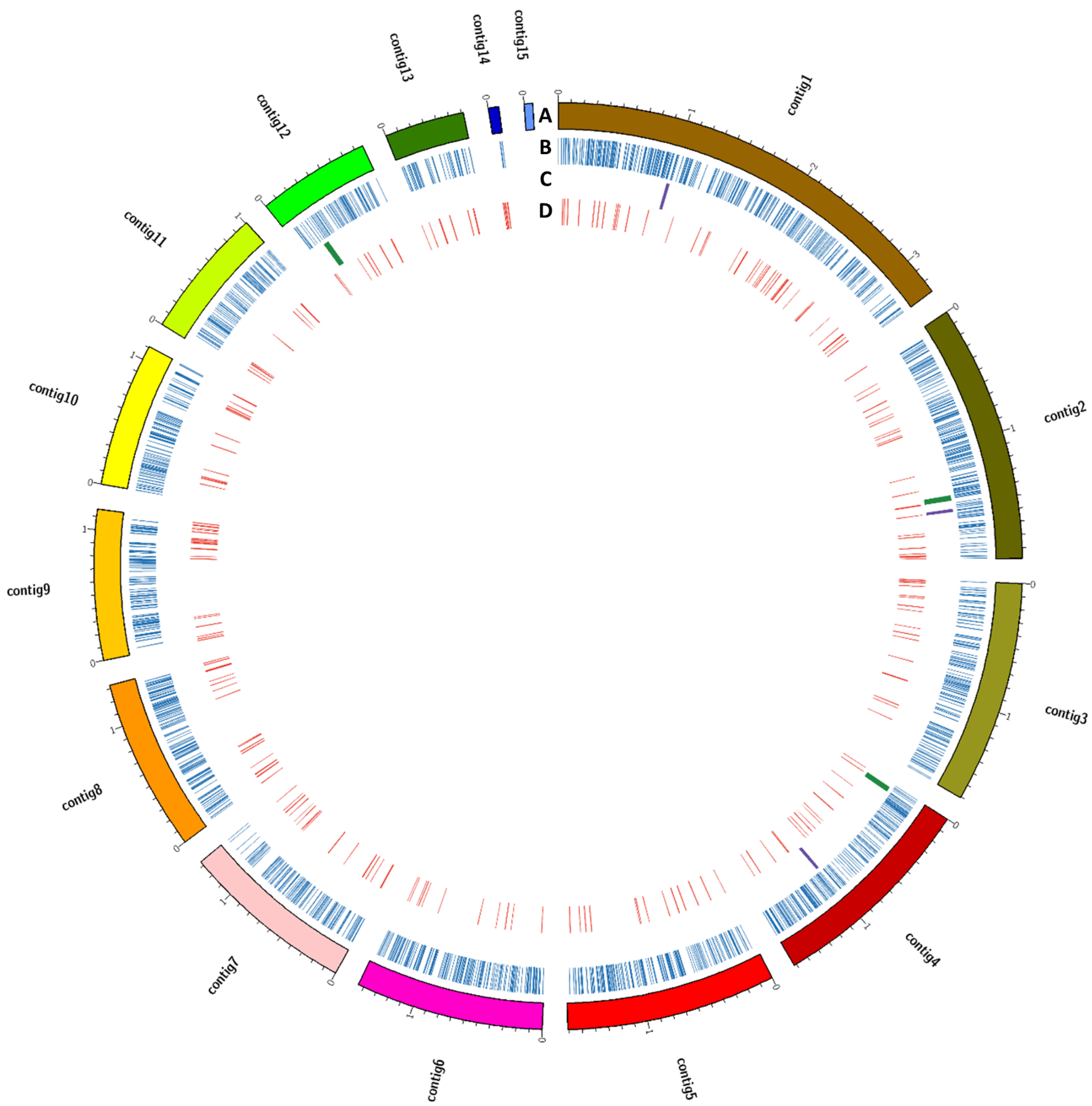

Figure S6. Circos plot of the strain 62UF-13 genome showing the genomic distribution of key functional features. From outer to inner, tracks represent: **(A)** contigs of strain 62UF-13 (minor ticks indicate 0.1 Mb; numbers denote the identifiers of the 15 contigs); **(B)** positions of genes encoding effector candidates (blue); **(C)** positions of secondary metabolite gene clusters. Colors indicate predicted cluster types: nonribosomal peptides (green), terpenes and *β*-lactones (purple), and other clusters (blue); **(D)** positions of genes encoding carbohydrate-active enzymes (CAZymes, red). This representation highlights the genome-wide organization and contig-level distribution of effector genes, secondary metabolite biosynthetic clusters, and CAZymes.

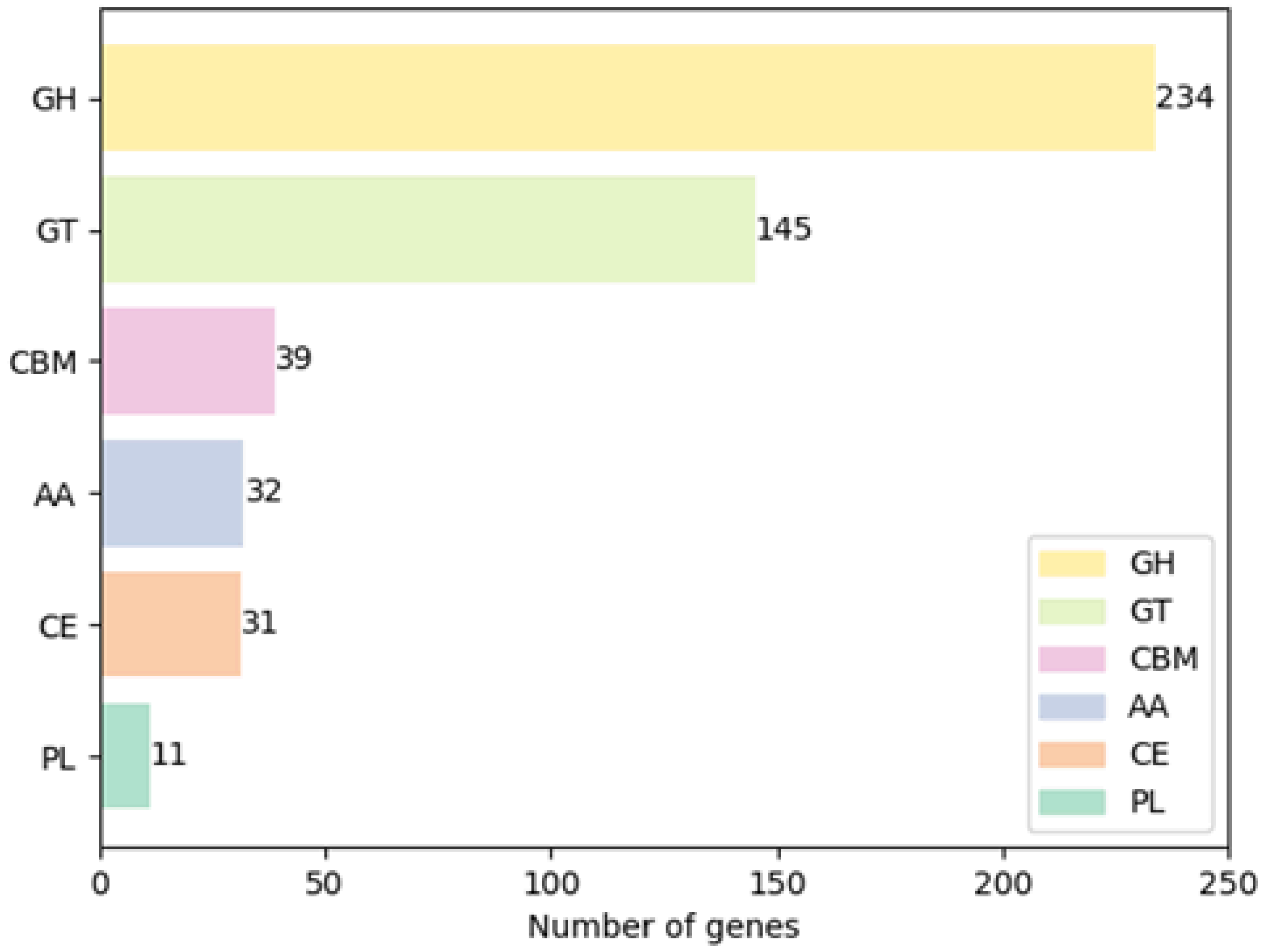

Figure S7. Distribution of carbohydrate-active enzymes (CAZymes) in the genome of strain 62UF-13. The number of genes predicted for each CAZyme family was determined using the dbCAN2 meta server. The genome contains a diverse repertoire of glycoside hydrolases (GH), glycosyltransferases (GT), carbohydrate-binding modules (CBM), carbohydrate esterases (CE), and auxiliary activities (AA), highlighting the metabolic versatility of the strain.
